# Integrin-specific hydrogels for growth factor-free vasculogenesis

**DOI:** 10.1038/s41536-022-00253-4

**Published:** 2022-09-27

**Authors:** Helena R. Moreira, Daniel B. Rodrigues, Sara Freitas-Ribeiro, Lucília P. da Silva, Alain da S. Morais, Mariana Jarnalo, Ricardo Horta, Rui L. Reis, Rogério P. Pirraco, Alexandra P. Marques

**Affiliations:** 1 3B’s Research Group, I3Bs – Research Institute on Biomaterials, Biodegradables and Biomimetics, University of Minho, Headquarters of the European Institute of Excellence on Tissue Engineering and Regenerative Medicine, Avepark – Zona Industrial da Gandra, Guimarães, 4805-017 Portugal; 2 ICVS/3B’s – PT Government Associate Laboratory, Braga/Guimarães, 4805-017 Portugal; 3 Department of Plastic and Reconstructive Surgery, and Burn Unity, Centro Hospitalar de São João, Porto, Portugal; 4 Faculty of Medicine - University of Porto, Porto, Portugal

**Keywords:** Stem-cell biotechnology, Biomaterials - cells, Regenerative medicine, Growth factor signalling

## Abstract

Integrin-binding biomaterials have been extensively evaluated for their capacity to enable de novo formation of capillary-like structures/vessels, ultimately supporting neovascularization in vivo. Yet, the role of integrins as vascular initiators in engineered materials is still not well understood. Here, we show that αvβ3 integrin-specific 3D matrices were able to retain PECAM1^+^ cells from the stromal vascular fraction (SVF) of adipose tissue, triggering vasculogenesis in vitro in the absence of extrinsic growth factors. Our results suggest that αvβ3-RGD-driven signaling in the formation of capillary-like structures prevents the activation of the caspase 8 pathway and activates the FAK/paxillin pathway, both responsible for endothelial cells (ECs) survival and migration. We also show that prevascularized αvβ3 integrin-specific constructs inosculate with the host vascular system fostering in vivo neovascularization. Overall, this work demonstrates the ability of the biomaterial to trigger vasculogenesis in an integrin-specific manner, by activating essential pathways for EC survival and migration within a self-regulatory growth factor microenvironment. This strategy represents an improvement to current vascularization routes for Tissue Engineering constructs, potentially enhancing their clinical applicability.

## Introduction

Vascularization of Tissue Engineering (TE) constructs is a pressing priority in the field since it is a crucial step to ensure construct viability in vivo. However, strategies capable of triggering controlled neotissue vascularization by vasculogenesis or angiogenesis are still elusive. Vasculogenesis is defined as the process in which blood vessels are generated in the absence of pre-existing ones^1^. During embryonic development, endothelial cells (ECs) differentiate from precursors termed angioblasts, assemble and form a de novo vascular plexus^2^. This process is orchestrated by a fine-tuned balance between different angio-regulatory growth factors^1^. In TE and regenerative medicine, biomaterials have been extensively evaluated regarding their capacity to support vasculogenesis^3,4^. Due to the recognition of biological cues by cell integrins, peptide-functionalized matrices have been used to independently promote adhesion of different cell types, including ECs. Functionalized peptide hydrogels with binding affinity for αvβ3/α5β1 integrins, for instance, support ECs survival and stimulate tube-like structure formation in vitro^5^. Others showed that ECs adhesion, migration, proliferation in vitro, and neovascularization in vivo was attained faster when cells specifically recognized α4β1 integrin-binding sites in functionalized scaffolds^6^. While it is assumed that integrins at the cell membrane are involved in these interactions with the biomaterials, a fundamental understanding of their engagement as vasculogenesis initiators is lacking.

Integrins such as β1 and β3, have been implicated in vasculogenesis. For instance, while loss of β1 integrin in nascent endothelium results in a disruption of ECs polarity and lumen formation^7^, inhibition of β3 integrin resulted in reduced migration, polarization, and proliferation of ECs^8^. Moreover, β1 and β3 integrins are known to be indispensable for the proper expression of VE-cadherin and therefore for tight cell–cell junction integrity, critical for generating non-leaky blood vessels^9,10^. Also, integrin β1-deficient vascular smooth muscle cells compromise the recruitment of mural cells for vessel stabilization leading to postnatal lethality in mice^11^. A wide variety of cells are known to highly express these integrin heterodimers and have been implicated in influencing and promoting the vasculogenic process. Such cell heterogeneity can be found in the stromal vascular fraction (SVF) of the adipose tissue^12,13^. Besides mesenchymal progenitors, the SVF comprises several other cell types including fibroblasts, pericytes, pre-adipocytes, ECs, and hematopoietic cells^14–16^. This complex cellular pool present in the SVF allows for the delivery of angiogenic growth factors in a self-regulating and dynamic manner that enhances the neovascularization of ischemic tissues^13^. Moreover, this angiogenic environment allows for complex and interconnected in vitro capillary-like networks in the absence of extrinsic growth factors^17^.

Taking into consideration the angiogenic properties of SVF cells and what is known about ECs response to peptide-modified materials, we hypothesized that integrin-specific 3D matrices are able to retain cells from the SVF capable of triggering and supporting vasculogenesis in vitro in the absence of extrinsic growth factors. Specific integrin engagement was confirmed as a vascular morphogenic signal within engineered matrices by activating essential pathways for EC survival and migration, in a self-regulatory growth factor microenvironment. Additionally, we provide evidence that triggering vasculogenesis in vitro benefits neovascularization in vivo, which guarantees engraftment and indicates that the proposed strategy can enhance the efficacy of TE constructs.

## Results

### Integrin-specific spongy-like hydrogels modulate vasculogenesis triggering

**Fig. 1:**
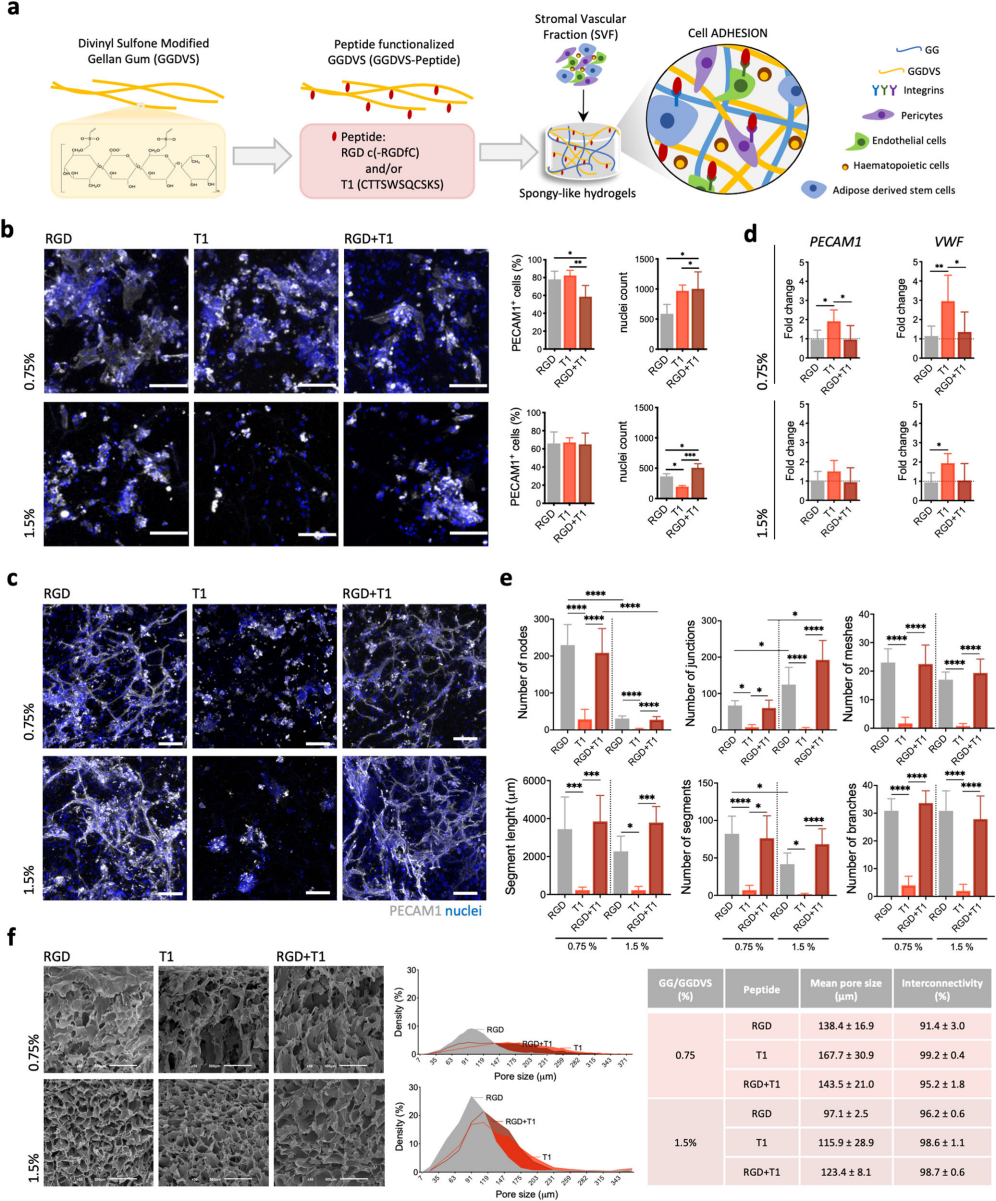
Integrin-specific spongy-like hydrogels modulate vasculogenesis triggering. **a** Schematic representation of the rationale of using GGDVS functionalized with T1 and RGD peptides to capture cells involved in vasculogenesis from the stromal vascular fraction (SVF) of adipose tissue. **b** Representative immunocytochemistry images of the expression of PECAM1 in SVF cells kept in the functionalized materials after 3 days of culture and respective quantification of the percentage of PECAM1^+^ cells and total number of cells. Scale bar = 100 µm. **c** Representative immunocytochemistry images of the organization of PECAM1^+^ cells in the functionalized materials after 7 days of culture. Scale bar = 100 µm. **d** *PECAM1* and *VWF* mRNA expression in cells kept in the functionalized materials at day 7. mRNA expression was determined by qPCR using *β2M* as reference gene and normalized to the respective expression at day 5 to understand the variation along time for each condition. **e** Quantification of the number of nodes, junctions, meshes, segments, branches, and segments length in the capillary-like structures formed after 7 days of culture. **f** Microstructural features (pore size and interconnectivity) of GG/GGDVS-peptides dried polymeric networks visualized by SEM and quantified by μ-CT. Scale bar = 100 µm. Quantitative results are expressed as the mean ± standard deviation where *n* = 5, *p < 0.05, **p < 0.01, ***p < 0.001, ****p < 0.0001, one-way or two-way ANOVA with Tukey multiple comparison post-test.

GG spongy-like hydrogels were designed to present integrin-specific peptides to SVF cells capable of triggering vasculogenesis (Fig. 1a). Using Michael-type addition chemistry, GG was functionalized with DVS to allow the conjugation of cysteine-terminated peptides via reaction with the thiol group. Two peptides with different integrin-binding specificities, cyclic RGD (RGD, cyclo-RGDfC) derived from fibronectin-III domain with high binding affinity for αvβ3 integrin^18^, and T1 (GQKCIVQTTSWSQCSKS) present in CCN1 (CYR61) III domain as an α6β1 integrin-binding peptide^19^, were conjugated to GGDVS with >89% conjugation efficiency (Supplementary Fig. 1a). A formulation containing both RGD and T1 with affinity for both αvβ3 and α6β1 integrins was also prepared. The integrin-specific spongy-like hydrogels were prepared as previously described^20^, combining the GGDVS-peptide with unmodified GG at the time of hydrogel formation to form a semi-interpenetrating network (Supplementary Fig. 1b). Two formulations with 0.75% and 1.5% total polymer concentration (Supplementary Table 1), were prepared to take into consideration the properties of the scaffolds, known to impact overall cellular behavior^21^.

Vasculogenic cells, such as endothelial progenitor and EC, highly express α6β1 integrin and αvβ3 integrin^22,23^. The populations retained in our spongy-like hydrogels are enriched in those cells, present in the SVF (Supplementary Fig. 2), as demonstrated by the number of PECAM1^+^ cells (Fig. 1b). After 3 days of culture, 80% (0.75% spongy) and 60% (1.5% spongy) of the captured cells were PECAM1^+^, for both RGD− and T1-presenting spongy-like hydrogels. Moreover, the simultaneous presentation of RGD and T1 led to a significantly lower percentage of PECAM1^+^ captured cells (60%) in comparison with single peptide-presenting materials, although only for those with a lower concentration of polymer. Interestingly, these results are independent of the total number of cells within each material since the total number of nuclei counts was significantly higher in the RGD+ T1-presenting spongy-like hydrogels than in the RGD and T1 counterparts. Moreover, while the number of PECAM1^+^ cells is similar in RGD− and T1-presenting spongy-like hydrogels, the total number of cells decreases with increased polymer concentration.

Knowing that αvβ3 has a key role in EC survival, migration, and differentiation^24^, and that the activation of α6β1 regulates endothelial tube formation^25^, we next assessed the ability of our integrin-specific spongy-like hydrogels to support SVF-derived ECs organization and formation of capillary-like structures in the absence of extrinsic angiogenic growth factors. The presence of PECAM1^+^ capillary-like structures was only observed in the RGD− or RGD+ T1-containing materials (Fig. 1c). The SVF cells in the T1-containing materials tended to cluster in roundish aggregates, similarly to what is observed in the control materials without peptide (Fig. 1c, Supplementary Fig. 3a). Interestingly, the expression of *PECAM1* and *VWF* was upregulated in the cells adhered to the T1-containing materials on day 7 (Fig. 1d, Supplementary Fig. 3b). The analysis of the capillary-like structures showed that the number of nodes, junctions, meshes, segments, branches, and segment length was significantly higher in the RGD-containing spongy-like hydrogels than in the T1 materials (Fig. 1e, Supplementary Fig. 4). No differences were observed between RGD− and RGD+ T1-modified materials, regardless of the polymer concentration. Nonetheless, the number of nodes and junctions respectively diminished and increased in the RGD and RGD+ T1 with higher polymer concentration. This might be related to a higher small pore density within the materials with a higher polymer amount (Fig. 1f), potentially enclosing higher cell densities at specific areas fostering the formation of junctions (four or more segments) rather than new nodes (three or more segments connected) formation.

**Fig. 2:**
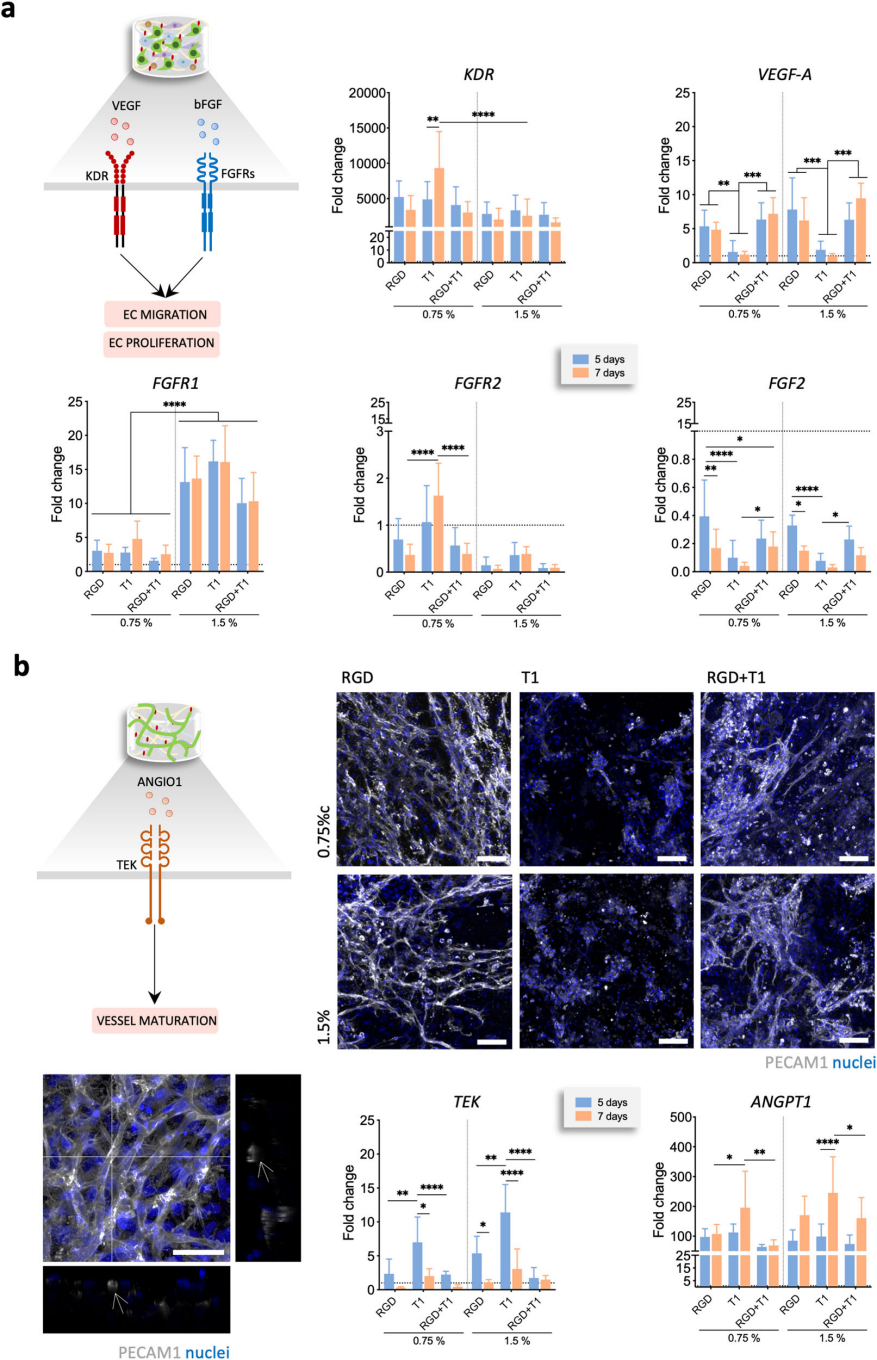
VEGF and FGF2 are involved in EC migration and proliferation that results in the formation of vascular structures. **a** Schematic representation of the hypothesized involvement of VEGF and FGF2 pathways. *KDR*, *VEGF*, *FGFR1*, *FGFR2,* and *FGF2* mRNA expression in cells cultured in the functionalized materials on day 5 and 7. mRNA expression was determined by qPCR using *β2M* as reference gene and normalized to the results for non-modified material on day 5. **b** Schematic representation of the hypothesized involvement of ANGPT1 pathway in vessel maturation. Representative immunocytochemistry images of SVF cells expressing PECAM1^+^ in the functionalized materials after 14 days of culture. Scale bar = 100 µm. Detail of the vascular-like network formed by PECAM1^+^ cells in the 0.75% GG/GGDVS-RGD spongy-like hydrogel after 14 days in culture showing several lumen (white arrows). Nuclei were counterstained with DAPI. Scale bar = 50 µm. *TEK* and *ANGPT1* mRNA expression in cells cultured in the functionalized materials on days 5 and 7. mRNA expression was determined by qPCR using *β2M* as reference gene and normalized to the results for non-modified material at day 5 to allow comparison among the conditions. Quantitative results are expressed as the mean ± standard deviation where *n* = 5, *p < 0.05, **p < 0.01, ***p < 0.001, ****p < 0.0001, two-way ANOVA with Tukey multiple comparison post-test.

Since vascular endothelial growth factor (VEGF) and basic fibroblast growth factor (FGF-2) pathways are key in promoting ECs migration and proliferation during vasculogenesis, we looked at the gene expression of *VEGF*, *FGF2* and respective receptors, *KDR* and *FGF receptor (FGFR) 1* and *2* to assess their involvement in the response to the functionalized materials. In RGD-containing materials where capillary-like structures were able to form, the angiogenic genes *VEGF* and *FGF2* were expressed in higher amounts than in T1-containing materials. In opposition, the respective receptors, *KDR* and *FGFR2*, but not *FGFR1*, were upregulated for the T1-containing materials (Fig. 2a). Both *FGF2* and *FGFR2* were downregulated in relation to the control material without peptide.

When the RGD-containing materials were kept in culture over 14 days, the capillary-like structures formed an intricate network within the whole structure in which lumens were clearly identified (Fig. 2b, Supplementary Fig. 5, Supplementary Video 1). Considering that the SVF also contains stromal cells capable of acting as mural cells, we then analyzed changes in the expression of angiopoietin 1 (*ANGPT1*) and its receptor (Tyrosine-protein kinase receptor, *TEK*) (Fig. 1c), known to be involved in pericyte recruitment and vessel maturation^26,27^. The expression of both *ANGPT1* and *TEK* was higher in the T1-containing materials, and the presence of RGD did not involve alterations in the expression of the *TEK* in comparison with the controls without peptides. These results were independent of polymer concentration.

Overall, our results indicate that RGD-mediated adhesion of PECAM1^+^ cells from the SVF triggers vasculogenesis and supports the formation and maturation of capillary-like structures in vitro, without the addition of extrinsic growth factors. Since the number of PECAM1^+^ cells was superior in RGD− than in RGD+ T1-containing materials, and the survival rate of SVF cells was significantly higher in the materials with lower amount of polymer, 0.75% GG/GGDVS-RGD spongy-like hydrogels were used in the subsequent assays.

### Vasculogenesis in integrin-specific spongy-like hydrogels is prompted by FAK/paxillin signaling while apoptosis is prevented

**Fig. 3:**
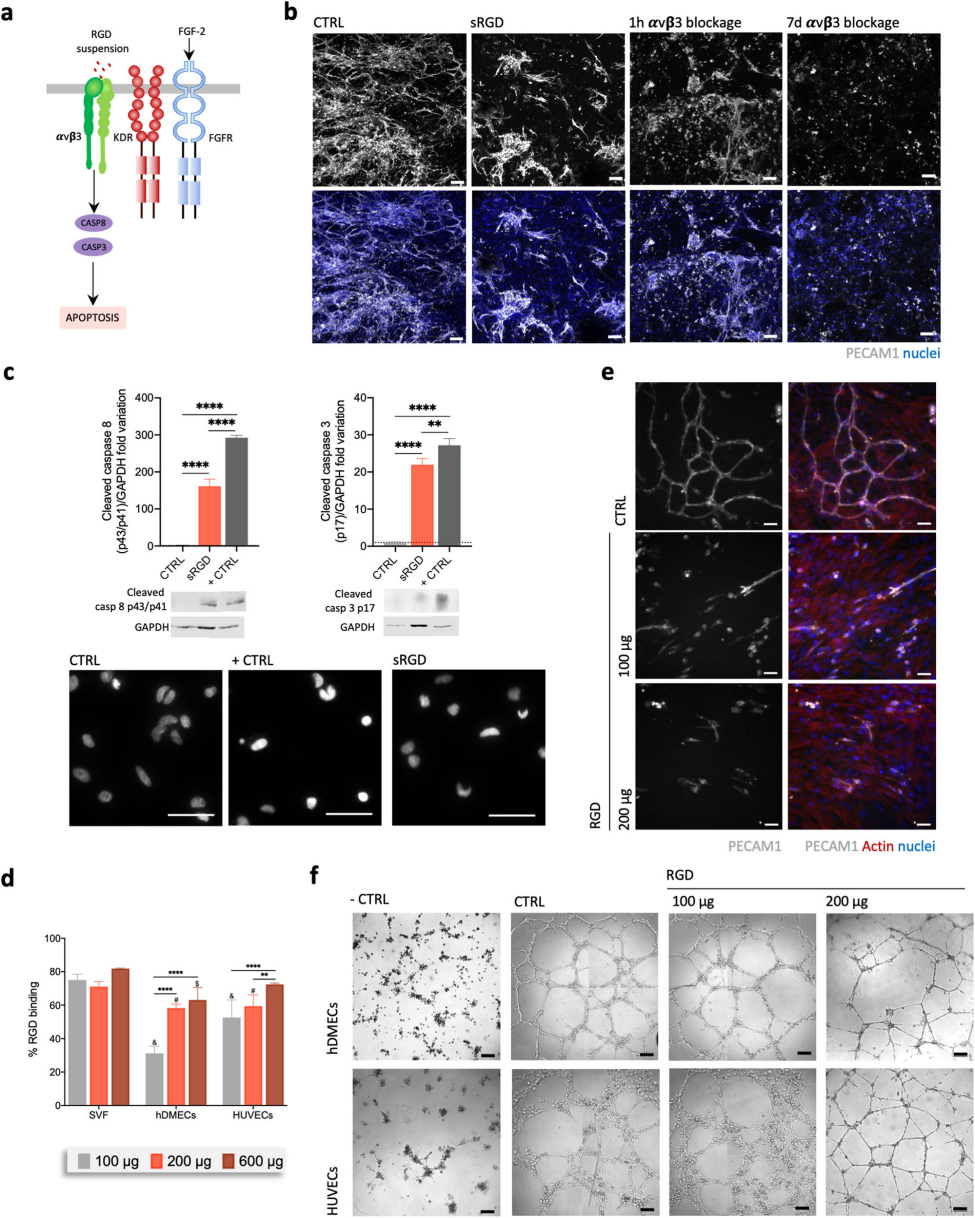
Integrin-specific spongy-like hydrogels prevent apoptosis of cells involved in the vasculogenic process. **a** Schematic representation of the studied apoptosis signaling pathway. **b** Representative images of the organization of SVF PECAM1^+^ cells after 7 days of culture in the 0.75% GG/GGDVS-RGD when pre-incubated with RGD peptide (sRGD) or with integrin αvβ3 blocking antibody for 1 h (1 h αvβ3 blockage), or in the presence of αvβ3 blocking antibody (7d αvβ3 blockage). Control (CTRL) refers to SVF cells directly seeded in the material. Scale bar = 100 µm. **c** Protein level of cleaved caspase 8 and cleaved caspase 3 in SVF cells 24 h after peptide exposure or mitomycin C (+CTRL) with correspondent representative western blot bands. Plotted western blot data was determined in relation to GAPDH expression. Representative nuclei images from SVF cells labeled with Picogreen 24 h after exposure to RGD peptide or mitomycin C (+CTRL). Scale bar = 50 µm. **d** Percentage of RGD peptide binding to SVF cells, hDMECs, and HUVECs after 30 min incubation. **e** Representative images of the organization of PECAM1^+^ SVF cells pre-incubated with different amounts of the RGD peptide and cultured on standard 2D surfaces for 7 days. Scale bar = 50 µm. **f** Representative images of hDMECs and HUVECs in a Matrigel assay, pre-incubated with different amounts of the RGD peptide or in their respective basal media without any angiogenic growth factors (negative control (−CTRL)). In the positive control condition (+CTRL), cells were cultured in their optimized media without pre-incubation with RGD peptide. Scale bar = 200 µm. Quantitative results are expressed as the mean ± standard deviation where *n* = 3, *p < 0.05, **p < 0.01, ***p < 0.001, ****p < 0.0001, one-way ANOVA with Tukey multiple comparison post-test or Kruskal–Wallis test with Dunn’s multiple comparison post-test.

Given our results confirming the triggering of vasculogenesis in vitro by αvβ3-specific spongy-like hydrogels, we wanted to understand the underlying mechanisms. To infer the involvement of VEGF and FGF2 pathways during the interaction of the SVF cells’ αvβ3 integrin with the RGD present in the spongy-like hydrogels, function-blocking peptide studies were carried out. When in suspension, cyclic RGD is known to act as an antagonist of its high-affinity binding integrins (αvβ3, αvβ5, or αIIIβ3), leading to cell apoptosis and suppressed angiogenesis^28,29^ (Fig. 3a). Thus, SVF cells were incubated with RGD in suspension prior to their seeding in the RGD-containing spongy-like hydrogels. The formation of capillary-like structures in the RGD-containing spongy-like hydrogels was impaired when SVF cells were pre-incubated with RGD (Fig. 3b). Moreover, the involvement of αvβ3 integrin was further confirmed by function-blocking antibody studies. Vasculogenesis was completely inhibited in the presence of a function-blocking antibody against αvβ3 over the course of a 7-day culture period in RGD-containing spongy-like hydrogels (Fig. 3b). An inhibition of capillary-like structure formation similar to what was observed when SVF cells were pre-incubated with RGD was seen when the blocking antibody was also incubated with cells prior to seeding, suggesting partial recovery of αvβ3 expression when the blockage was removed. These results demonstrate the specificity of the SVF vasculogenic cells interaction with the materials via αvβ3 integrin. Moreover, we further confirmed the activation of the apoptosis cascade by caspase 8 and caspase 3 cleavage when cells were pre-incubated with RGD (Fig. 3c, Supplementary Fig. 6). The amount of RGD peptide (100 μg) was defined based on the binding efficiency (Fig. 3d), as well as on the impaired capacity of the cells to form vascular-like structures in SVF (Fig. 3e) and in Matrigel (control micro and macrovascular ECs) (Fig. 3f). These results suggest the involvement of αvβ3 and of RGD-driven signaling in the formation of the capillary-like structures in our materials, preventing the activation of the caspase 8 pathway.

**Fig. 4:**
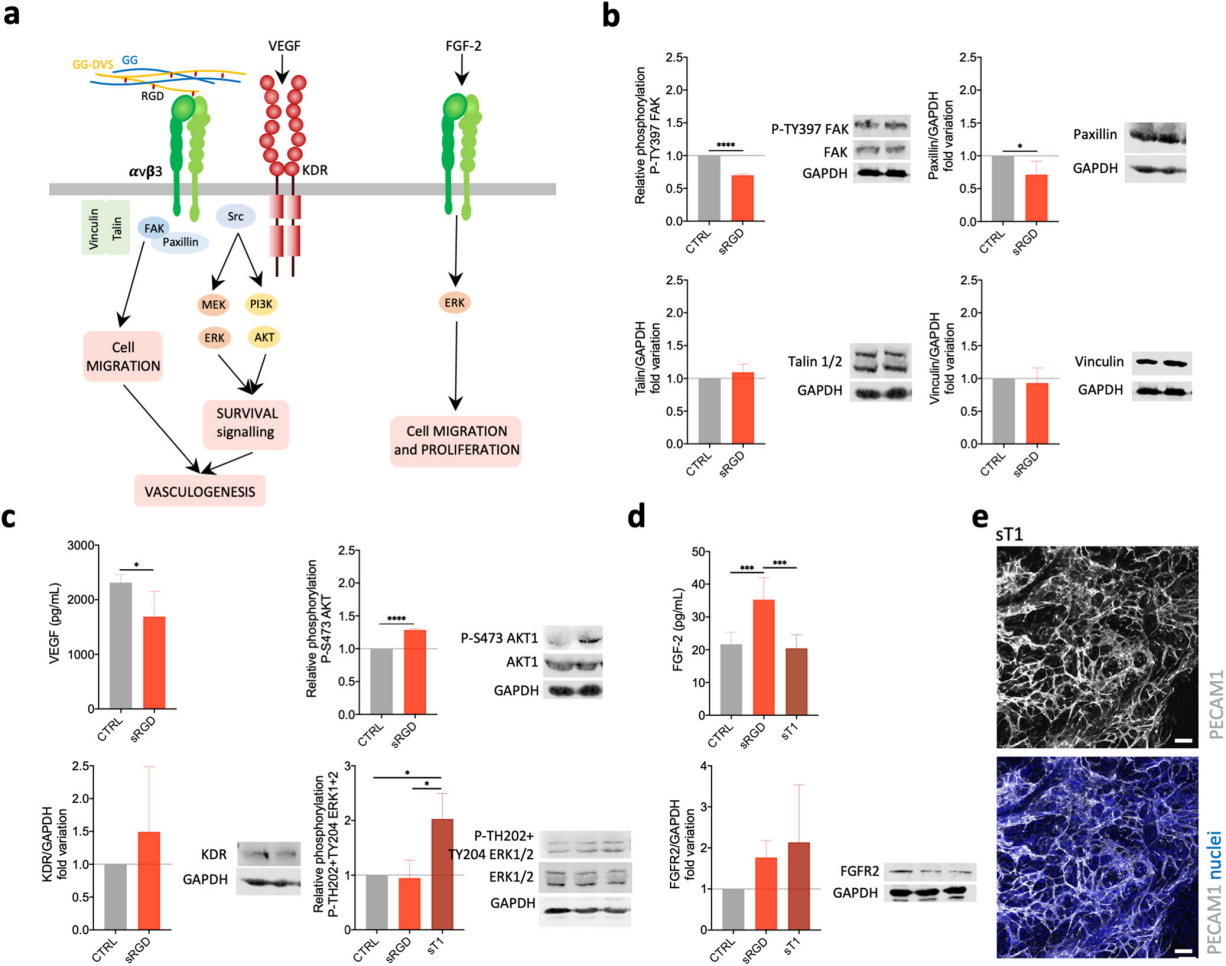
Involvement of VEGF and FGF2 pathways in the vasculogenesis in integrin-specific spongy-like hydrogels. **a** Schematic representation of the studied signaling pathways. **b** Expression of P-TY397 FAK, FAK, Paxillin, Talin 1 + 2. **c** VEGF secretion, and KDR, P-TH202-TY204 ERK1/2, ERK1/2, P-S473 AKT1, and AKT1 expression. Expression of the proteins (**b**, **c**) was determined by western blot in SVF cells, with and without pre-incubation with RGD peptide, cultured in the 0.75% GG/GGDVS-RGD materials for 7 days. Expression of P-TH202-TY204 ERK1/2, ERK1/2 was also determined when SVF cells were pre-incubation with T1 peptides. **d** FGF-2 secretion and FGFR2 expression determined by western blot in SVF cells, with and without pre-incubation with RGD and T1 peptides, cultured in the 0.75% GG/GGDVS-RGD materials for 7 days. All plotted western blot data was determined in relation to GAPDH expression. **e** Representative images of the organization of SVF PECAM1^+^ cells after 7 days of culture in the 0.75% GG/GGDVS-RGD when pre-incubated with T1 peptide. Results are expressed as the mean ± standard deviation where *n* = 3, *p < 0.05, **p < 0.01, ***p < 0.001, ****p < 0.0001, one-way ANOVA with Tukey multiple comparison post-test or two-tailed unpaired *t* test.

We next assessed which pathways were activated due to this integrin-ligand interaction. The focal adhesion kinase (FAK) is the main transducer of the integrin-mediated signaling pathway required for EC adhesion and motility, using paxillin as a major substrate for focal adhesion turnover during cell migration^30^ (Fig. 4a). When αvβ3 in SVF cells was blocked by the RGD peptide, phosphorylated FAK as well as paxillin were significantly downregulated, indicating an interference in the FAK/paxillin signaling response (Fig. 4b). Moreover, no differences were found in both talin 1/2 and vinculin, which seems to confirm the lack of integrin engagement and of the subsequent formation of focal adhesion complexes that require those proteins^31,32^. Migration and survival of ECs, and tube-like formation, are also regulated through signaling triggered by the crosstalk between αvβ3 integrin and KDR after VEGF binding^33–35^. In particular, the VEGF-A/KDR signaling cascade leads to the partial activation of the phosphatidylinositol 3-kinase (PI3K)/AKT and mitogen-activated protein kinase (MAPK)/extracellular-signal-regulated kinase-1/2 (ERK1/2) signal transduction pathways^36^. The pre-incubation with RGD peptide resulted in a decreased amount of secreted VEGF, although without alteration in the expression and quantity of its receptor KDR (Fig. 4c, Supplementary Fig. 7a). Moreover, although AKT1 phosphorylation was upregulated, phospho-ERK1/2 was not altered, which seems to indicate that VEGF signaling might be contributing to some extent to the observed response.

On the other hand, it is known that FGF-2 depends on αvβ3 integrin to initiate angiogenesis^37^. Upon binding of the FGF-2 to αvβ3 integrin, ERK1/2 phosphorylation occurs inducing EC adhesion and proliferation^38^. Moreover, when this integrin is blocked, the produced FGF-2 binds to the FGFRs which is not sufficient to induce EC proliferation^38^. The pre-incubation of SVF cells with RGD peptide did not influence ERK1/2 phosphorylation but resulted in an increased amount of secreted FGF-2 and FGFR2, while no differences were observed for the FGFR1 receptor (Fig. 4d, Supplementary Fig. 7a, b). In opposition, a significantly higher ERK1/2 phosphorylation (Fig. 4c), accompanied by the formation of capillary-like structures (Fig. 4e), was observed when SVF cells were pre-incubated with the T1 peptide which blocks the α6β1 integrins. This seems to confirm that the interaction of FGF-2 with αvβ3 integrin occurs at a significantly higher level when α6β1 integrin is blocked and thus might not be predominant in triggering vasculogenesis in our materials.

### Preformed human capillaries foster neovascularization in vivo

**Fig. 5:**
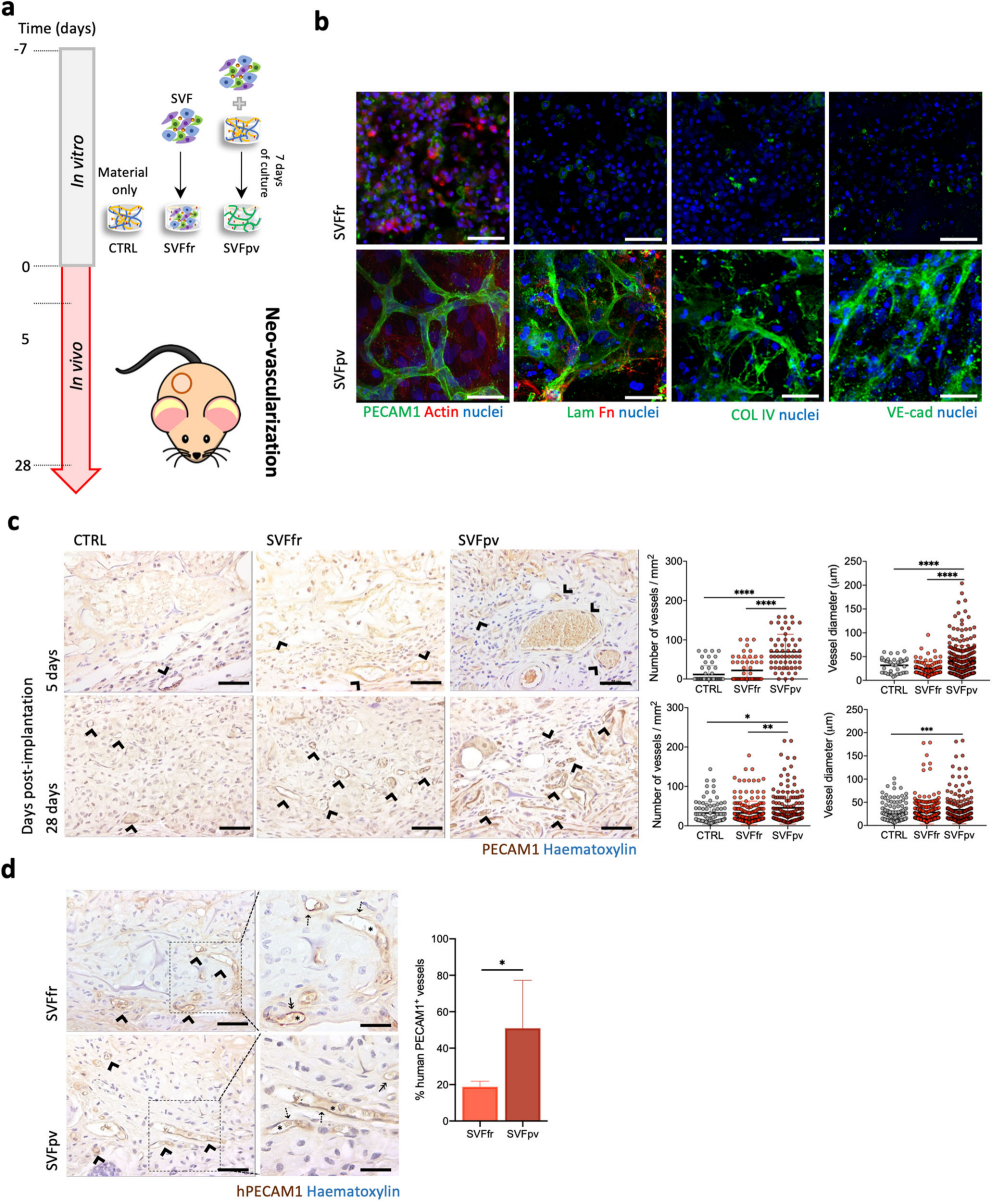
Preformed human capillaries foster neovascularization in vivo. **a** Schematic representation of the test conditions: CTRL (GG/GGDVS-RGD spongy-like hydrogels without cells), SVFfr (freshly isolated SVF seeded in the material), SVFpv (SVF seeded in the material and cultured for 7 days in vitro prior to implantation). **b** Representative immunocytochemistry images of the expression of PECAM1 (anti-human), Collagen type IV (COL4), Laminin (Lam), Fibronectin (Fn), VE-cadherin (VE-cad), and F-actin in SVF cells seeded in the 0.75% GG/GGDVS-RGD spongy-like hydrogels, without (SVFfr) and after 7 days of vitro pre-culture (SVFpv). Nuclei were counterstained with DAPI (nuclei). Scale bar = 50 µm. **c** Representative immunocytochemistry images showing PECAM1^+^ vessels (arrowhead, anti-mouse and anti-human) at the transplantation site on days 5 and 28, and respective quantification of the number of vessels and diameter. **d** Representative immunocytochemistry images of vessels incorporating human PECAM1^+^ cells (arrowhead, anti-human) at the transplantation site on day 28 and respective quantification. Higher magnification images show chimeric (⇡) and human (↟) vessels containing mouse erythrocytes (*). Scale bar = 500 µm, 100 µm, 50 µm. Quantitative results are expressed as the mean ± standard deviation, *p < 0.05, **p < 0.01, ***p < 0.001, ****p < 0.0001, Kruskal–Wallis test with Dunn’s multiple comparison post-test or two-tailed unpaired *t* test.

Prevascular networks have been shown to accelerate the vascularization of 3D tissue-engineered constructs and enhance their ability to anastomose with the host’s vascular networks^39^. We wanted to understand if the integrin specificity of the spongy-like hydrogels would be enough to overcome the need for an in vitro prevascularization step to ensure in vivo neovascularization. For that, freshly isolated SVF cells were seeded in the material and immediately implanted (SVFfr) or cultured for 7 days in vitro prior to implantation (SVFpv) (Fig. 5a). In the latter condition, PECAM1^+^ cells of the SVF were able, in vitro, to promote homotypic interactions through VE-cadherin and form stable capillary-like structures characterized by the presence of basement membrane proteins such as laminin and collagen type IV (Fig. 5b).

Given the relevance of determining if prevascularization of the constructs contributed to faster vascularization, we assessed the total number of vessels and vessel diameter at the implantation site as early as 5 days post-transplantation (Fig. 5c, Supplementary Fig. 8a). The number of vessels in the prevascularized group was significantly higher than in the control and non-prevascularized groups. The vessel diameter was also significantly higher than in the non-prevascularized group, but not different from the control. After 28 days of transplantation, both the number and diameter of vessels were significantly higher in the prevascularized group.

We further inquired about the fate of the human PECAM1^+^ cells from the SVF and their involvement in neotissue vascularization. Vessels with human PECAM1^+^ cells were identified in both experimental groups (Fig. 5d, Supplementary Fig. 8b) but at a significantly higher percentage in the prevascularized condition. Moreover, both chimeric and human PECAM1^+^ vessels contained mouse erythrocytes in their lumen (Fig. 5d), evidencing the functionality of the in vitro-formed capillary-like structures. These results indicate that the prevascularization of the constructs was essential to foster vascularization and the connection with the host circulatory system.

## Discussion

Strategies toward in vitro vasculogenesis are a challenging and pressing priority in TE. Biomaterials with integrin-binding specificities have been extensively evaluated for their capacity to enable de novo formation of capillary-like structures/vessels and ultimately support neovascularization, but the role of integrins as vascular initiators in engineered materials is poorly understood. Considering this, we used integrin-specific biomaterials to understand whether the response of SVF cells would be capable of triggering vasculogenesis.

Taking into consideration the knowledge of ECs responses to peptide-modified materials, different peptides targeting αvβ3 and α6β1 integrins were used to engineer spongy-like hydrogels. In the 3D microenvironment, we showed that RGD spongy-like hydrogels (αvβ3 integrin ligand), but not T1 (α6β1 integrin ligand), support the spontaneous formation of a microvascular network in vitro by SVF cells. Moreover, SVF cells response was similar in both RGD+T1 spongy-like hydrogels and RGD-only spongy-like hydrogels. This suggests that the presence of T1 peptide does not negatively interfere with the formation of the capillary-like structures. Interestingly, this formation is independent of spongy-like hydrogel’s capacity to retain PECAM1^+^ cells from the SVF since the number of PECAM1^+^ cells in the RGD+T1-containing materials is significantly lower than in the other structures. Thus, it seems that the content of angiogenic cells in the SVF in conjunction with the proposed materials supports a reduction of the initial cell number without compromising the triggering of the vasculogenesis, which might be beneficial in the clinical context. Moreover, considering these distinct findings, the data reported herein strongly supports the view that ECs in the SVF require binding and activation of αvβ3 integrin, but not α6β1, to start vasculogenesis. In the presence of a function-blocking antibody against αvβ3 integrin, the capillary-like structure formation was completely inhibited in the RGD−containing materials. When the blockage was limited to a partial time frame, some capillary-like structures were observed, suggesting some recovery of the αvβ3 integrin expression along the culture. These results were further confirmed by using αvβ3 integrin-specific soluble RGD peptides. Thus, while scrambled RGD experiments would further strengthen our conclusions, we believe the two types of αvβ3-specific blocking studies are sufficiently demonstrative of the integrin specificity of the observed results. In addition to this, it was clear that no capillary-like structures were observed in the T1-only materials that interact with an integrin other than αvβ3. The T1 peptide ligand, α6β1 integrin, is highly expressed by pericytes and PECAM1^+^ cells, the latter also expressing αvβ3 integrin^23^. This dual linkage possibility provided by T1 could be linked to the positive modulation of some of the markers (*PECAM1*, *VWF*, *FGFR2*, *TEK*, and *ANGPT1*) in the T1-containing materials. However, we showed that it is the interaction of the αvβ3 integrin with the RGD present in the spongy-like hydrogels that are responsible for the capillary-like structure formation, potentially due to an effect on ECs survival and migration. Upon blocking the αvβ3 integrin, SVF cells entered the apoptosis cascade as demonstrated by the increase in cleaved caspase 8 and cleaved caspase 3. Considering the above we can strongly suggest that SVF PECAM1^+^ cells respond to the vasculogenic signal provided by the RGD peptide in our biomaterials. This is in agreement with what has been already observed regarding both progenitor and mature ECs expressing elevated levels of αvβ3 integrin^22,23^, that this integrin has a high affinity to the RGD moiety used in this study^18^ and ultimately that the RGD-αvβ3 integrin interaction is key to vasculogenesis^40^. Moreover, phosphorylated FAK, the main transducer of the integrin-binding with the ECM^30^, and paxillin, a signal transduction adapter protein associated with focal adhesions and one of the major substrates of FAK^41^, were downregulated. Other works have shown that the downregulation of those molecules is associated with impaired ECs motility, migration, and spreading^42^.

The cell complexity present in the SVF allows for a self-regulating dynamic of angiogenic and vasculogenic growth factors secretion^13^, including VEGF and FGF-2. Both are known to modulate vasculogenesis by promoting activation of downstream pathways associated to the αvβ3 integrin^43^. Therefore, those factors could be involved in the vasculogenesis observed in our system. When VEGF binds to KDR and the αvβ3 integrin is engaged with the ECM, αvβ3 integrin and KDR form a physical complex and synergize to regulate several cellular processes. These include maximal transduction of angiogenic growth factors, and tube formation^33–35^, via activation of the ERK1/2 and PI3K/Akt signal transduction pathways^36,44^. Our study demonstrated that after αvβ3 integrin blockage, the production of VEGF was downregulated although without alteration in the expression of its receptor KDR. Moreover, Akt was upregulated while no differences were observed for the phosphorylation of the ERK1/2 when αvβ3 integrin was blocked. Thus, the reduced secretion of VEGF might be linked to the overall need of cells to avoid apoptosis, as previously shown with the blockage of the αvβ3 integrin in ECs^45^, which in turn is in agreement with the activation of the Akt pathway, a critical regulator of PI3K-mediated cell survival^46^. While we do not demonstrate the direct involvement of VEGF, when the formation of VEGF-dependent vessels in a co-culture of pericytes and ECs is suppressed, pericytes activate the FGF pathway and start to express high levels of FGF-2 in a juxtacrine manner^47^. Our results are in agreement with this since FGF-2, potentially produced by other SVF cells, and the receptor FGFR2 were significantly upregulated when the αvβ3 integrin was blocked. However, it is also known that FGF-2/FGFRs-mediated signaling is not sufficient to induce EC migration, and binding of FGF-2 to αvβ3 integrin is necessary, promoting ERK1/2 phosphorylation^38^. The levels of phosphorylated ERK1/2 did not vary with the blocking of αvβ3 integrins in the SVF cells, despite the suppression of the formation of capillary-like structures. In opposition, when α6β1 integrins were blocked (T1 soluble peptide) this effect was reverted, and the levels of phosphorylated ERK1/2 were significantly upregulated. Therefore, our results seem to indicate that FGF-2-αvβ3 integrin interaction occurs at a significantly higher level when α6β1 integrin is blocked and thus might not be predominant in triggering the vasculogenesis in our αvβ3 integrin-specific 3D matrices. Thus, vasculogenesis in our αvβ3 integrin-specific biomaterials does not seem to be primarily linked to the self-regulating dynamic of the growth factors produced by SVF cells.

As a second goal, we wanted to validate our integrated TE approach as promoter of neovascularization by triggering vasculogenesis in the absence of extrinsic growth factors. For this, we compared prevascularized with non-prevascularized constructs in terms of engraftment and reparative activities in a skin wound healing model. The prevascular network formed in the αvβ3 integrin-specific materials is stable and mature, as shown by the presence of the basement membrane proteins and the involvement of VE-cadherin in cell–cell interaction. According to previous works^48–52^, prevascular networks can lead to improved anastomosis with the host blood vessels after transplantation, promoting neotissue formation. Our results after implantation of the prevascularized materials in a full-thickness skin wound are in agreement with those reports. Moreover, the presence of human-origin vessels perfused with mouse erythrocytes confirmed that the vascular network formed in vitro was able to inosculate with the host vasculature.

Overall, we demonstrate the αvβ3 integrin-specific biomaterials ability to trigger vasculogenesis by activating essential pathways for EC survival and migration, in a growth factor self-regulatory microenvironment. Moreover, triggering vasculogenesis in vitro benefits the engraftment of the prevascularized integrin-specific constructs indicating that this strategy can enhance the efficacy of tissue-engineered constructs.

## Methods

### Gellan gum (GG) chemical modifications and characterization

Gelzan powder (0.25% (w/v), Sigma, USA) was dissolved in deionized water (DI), under stirring at 90 °C. After dissolution, the temperature was lowered to room temperature (RT) and the pH adjusted to 12. Vinyl sulfone moieties (DVS, Sigma, USA) was added in excess (molar ratio of 30:1) to the GG solution and left to react for 1 h under stirring. Gellan gum-divinyl sulfone (GGDVS) was purified by three series of precipitation in cold diethyl ether (1:5) and dialysis against DI water for 3 days at 37 °C. The purified GGDVS was freeze-dried for further use.

GGDVS was dissolved in ultra-pure (UP) water (pH 8) at RT. Thiol-cyclo-RGD peptide (RGD, Cyclo(-RGDfC), >95% purity, GeneCust Europe) and T1 peptide (CTTSWSQCSKS, >95% purity, GeneCust Europe) were dissolved in UP water. A peptide solution (800 μM) was added to GGDVS solution (0.25-0.5% (w/v)) and left to react for 1 h at RT under agitation. After dissolution, GGDVS-peptide solution was dialyzed against UP water for 3 days and freeze-dried. The amount of peptide conjugated to the GGDVS polymer was quantified using micro-bicinchoninic acid assay (BCA) assay according to the manufacturers’ instructions (Fisher Scientific, USA). Calibration curves were prepared with the respective peptide (ranging from 0 to 200 µg mL^−1^), and GGDVS-RGD and GGDVS-T1 (ranging from 0.5 and 1 mg mL^−1^) solutions.

### GG-based spongy-like hydrogels fabrication

GG/GGDVS-peptide spongy-like hydrogels were prepared similarly to GG spongy-like hydrogels^20,21,53^ with modifications. A solution of GGDVS-peptide (0.25–0.5% (w/v) with 800 μM of peptide) was prepared for 1 h at RT. Meanwhile, a GG solution (0.5–1% (w/v)) was prepared at 90 °C for 30 min and allowed to reach 40 °C afterwards. Hydrogels were prepared by mixing the GGDVS-peptide solution with the GG solution (final concentration 400 μM). The dual peptide hydrogels were prepared by mixing, GGDVS-RGD:GGDVS-T1 solutions (1:1) with the GG solution. Hydrogels were cast into desired molds, frozen at −80 °C overnight, and then freeze-dried (Telstar, Spain) for 24 h to obtain GG/GGDVS-peptide(s) dried polymeric networks. Spongy-like hydrogels were formed after rehydration of the dried polymeric networks (Supplementary Table 1).

### Scanning electron microscopy

Scanning electron microscopy (SEM) was used to analyze the microstructure of the dried polymeric networks. Prior toanalysis, samples were sputter coated with a mixture of gold–palladium. A JSM-6010LV (JEOL, Akishima, Japan) microscope, operating with an accelerating voltage of 15 kV was used to capture images.

### Micro-computed tomography (μ-CT)

The dried polymeric structures’ microarchitecture was analyzed using a high-resolution X-ray microtomography system SkyScan 1072 scanner (SkyScan, Kontich, Belgium). Samples were scanned in high-resolution mode using a pixel size of 11.31 μm (magnification of ×23.30) and an integration time of 1.7 s. The x-ray source was set at 35 keV of energy and 215 μA of current. Representative datasets of 150 slices were transformed into a binary picture using a dynamic threshold of 45e225 (gray values) to distinguish polymer material from pore voids. Pore size and interconnectivity were obtained using CT Analyzer software (v1.5.1.5, SkyScan).

### SVF isolation and characterization

Adipose tissue was harvested from lipoaspirates or fat tissue from skin specimens of healthy donors (IMC 20.8–26.8) undergoing abdominoplasties after written informed consent and under the protocol established and approved between the Ethical Committees of Hospital S. João (Porto, Portugal) (Nr 477/2020) and University of Minho (CEICVS Nr 135/2020). Adipose tissue was digested with Collagenase type II (0.05% (w/v), Sigma, USA) under agitation for 45 min at 37 °C. SVF was obtained after filtration and centrifugation (800 × *g*, 10 min, 4 °C). SVF pellet was resuspended in red blood cell lysis buffer (155 mм of ammonium chloride, 12 mм of potassium bicarbonate, and 0.1 mм of ethylenediamine tetraacetic acid (EDTA), all from Sigma-Aldrich, Germany) and incubated for 10 min at RT. After centrifugation (300 × *g*, 5 min, RT), the supernatant was discarded, and the cell pellet was re-suspended for immediate use.

SVF characterization was performed using flow cytometry regarding the expression of surface cell markers CD105, CD73, CD90, CD45, CD34, PECAM1, and CD146. Furthermore, cells were labeled with DRAQ5 (eBioscoence, USA) for nuclear staining to discern the cells of interest from any remaining erythrocytes and tissue debris (Supplementary Fig. 9). Flow cytometry was conducted right after isolation for each biological sample. The referred antibodies (Supplementary Table 2) were added to 2.5 × 10^5^ cells, incubated according to the manufacturer’s concentrations for 20 min at RT, washed with PBS, and resuspended in PBS with 1% (v/v) Formalin (BioOptica, Italy). 2 × 10^4^ events were acquired in a BD FACSCalibur (BD Biosciences, USA) and analyzed using the Cyflogic software (v1.2.1, Finland).

### EC isolation and culture

Human dermal microvascular endothelial cells (hDMECs) were harvested from human skin samples obtained from abdominoplasties after written informed consent and under the protocol mentioned before. Briefly, skin specimens were cut into small fragments and incubated overnight in dispase (2.4 U mL^−1^) (BD Biosciences, USA) at 4 °C. hDMECs were obtained through the filtration and centrifugation of the dispase solution. hDMECs were cultured in gelatin (0.7% (w/v), Sigma, USA) coated flasks with EGM-2 MV (Lonza, USA). Cells were passaged at 70–90% confluence and used at passage 3–4.

Human umbilical vein endothelial cells (HUVECs) were purchased from Lonza (USA) and routinely cultured under the conditions defined by the company on gelatin type A (0.7% (w/v)) coated plates and EndoGRO-VEGF Complete Media Kit (Millipore, USA). Cells were passaged at 70–90% confluence and expanded up to passage 6 for all experiments.

### Peptide-integrin-binding assay

SVF cells were pre-incubated with RGD peptide (100 μg, 200 μg, or 600 μg) in suspension in minimal essential medium (α-MEM, Gibco, USA) without FBS for 30 min at 37 °C. The amount of peptide in the supernatant was quantified using micro-BCA assay according to manufacturers’ instructions and the percentage of peptide-integrin-binding was determined in relation to the initial quantity.

### Function-blocking peptide studies

SVF cells were pre-incubated with (i) RGD peptide (100 μg or 200 μg) in suspension in α-MEM without FBS for 30 min at 37 °C, (ii) anti-integrin αvβ3 (10 μg mL^−1^, clone LM609, MAB1976, Millipore, Portugal) in α-MEM without FBS for 1 h at 4 °C or (iii) anti-integrin αvβ3 for 1 h at 4 °C plus 7 day period in 37 °C. Afterwards, cells were seeded at a density of 1.5 × 10^6^ cells in six-well plates and further cultured for 7 days in complete α-MEM media before analysis of the organization of the cells.

For the hDMECs (micro ECs) and HUVECs (macro ECs), cells were pre-incubated with the same amounts of RGD peptide in their respective media without growth factors or FBS prior plating on Matrigel. Matrigel (Corning, USA) was added to 96-well plates and kept in a humidified incubator for 30 min prior seeding the hDMECs or HUVECs at a density of 1.5 × 10^4^ cells/well. The organization of the cells into capillary-like structures was assessed after 24 h of culture in a humidified incubator at 37 °C, 5% of CO_2_.

Micrographs were taken using an Axio Observer inverted Microscope (Zeiss, Germany) with the ZEN Blue 3.2 software (Zeiss, Germany).

### Apoptosis assay

SVF cells were pre-incubated with RGD (100 μg) in suspension or with mitomycin C (100 μg mL^−1^, Sigma, Portugal) as a positive control of apoptosis, prior seeding at a density of 1.5 × 10^6^ cells/well in a six-well plate. After 24 h, cells were collected for western blot analysis or stained with Picogreen (1:1000, Invitrogen, USA) for 20 min at RT. Nuclei images were taken using an Axio Observer inverted Microscope with the ZEN Blue 3.2 software.

### Cell-laden GG/GGDVS-peptide(s) spongy-like hydrogels

A SVF cell suspension containing 1.5 × 10^6^ cells was prepared in 30 μL of α-MEM supplemented with fetal bovine serum (10% (v/v), FBS, Invitrogen, USA) and antibiotic/antimycotic solution (1% (v/v), Invitrogen, USA) and dispensed dropwise on the top of the dried polymeric networks. Constructs were incubated for 30 min, at 37 °C, 5% CO_2_ to allow maximum cell entrapment within the structures and then fresh medium was added up to a total volume of 1 mL. For the function-blocking peptide study, SVF cells were pre-incubated with RGD (100 μg) or T1 (200 μg) in suspension prior seeding.

### Total RNA extraction and cDNA synthesis

Constructs were collected in Tri-reagent (400 μL, Sigma-Aldrich, Portugal) and preserved at −80 °C. For RNA extraction, samples were thawed, and chloroform (80 μL, Sigma-Aldrich, Portugal) was added. Following an incubation period of 15 min at RT, samples were centrifuged at 4 °C for 20 min at 12,000 rpm. The aqueous phase was then collected and isopropanol (200 μL, VWR, Portugal) were added. Samples were further incubated for 10 min at RT and then centrifuged at 4 °C for 10 min at 12,000 rpm. The supernatants were discarded, and the pellets were washed once with 100% ethanol and twice with 70% ethanol, by centrifugation at 4 °C for 5 min each at 12,000 rpm. Extracted RNA was kept in RNAse/DNAse-free water (10 μL, Lonza, Belgium).

RNA quantity and purity were assessed using a NanoDrop N-1000 Spectrophotometer (Thermo Fischer Scientific, USA). Samples with a 260/280 nm ratio between 1.6 and 2.2 were used for cDNA synthesis. Synthesis was performed using a QSCript cDNA SuperMix (Quanta Biosciences, USA) and a reverse transcription polymerase chain reaction (RT-PCR) Mastercycler (Eppendorf, Germany). An initial amount of 1 μg of RNA in RNAse/DNAse free water was used for a total volume of 20 μL.

### Quantitative real-time PCR (qPCR)

qPCR was used to detect the expression of vasculogenesis-associated genes (Supplementary Table 3). Candidate primers and the reference beta-2 microglobulin (β2M) genes were designed using the Primer-Blast database (NCBI, USA). For qPCR reactions, synthesized cDNA (1 μl) was used in a 20 μl reaction containing PerfeCTa® SYBR Green FastMix (10 μl, Quanta Biosciences, USA) and sense and antisense primers (300 nм) using a MasterCycler Realplex4 (Eppendorf, Germany). Reaction conditions comprised an initial 2 min denaturation step at 95 °C, followed by 45 cycles of 95 °C denaturations for 10 s, a 30 s annealing step at the required temperature (Supplementary Table 3), and a 10 s elongation step at 72 °C. Transcripts abundances were normalized to the expression of β2M. Samples were run in triplicate in each assay. Normalized expression values were calculated following the mathematical model proposed by Pfaffl using the formula: 2^−ΔΔCt ^^54^.

### Western blot

Cell-laden spongy-like hydrogels were collected in loading buffer (0.05% (w/v) bromophenol blue, glycerol (30% (v/v)), 5 M EDTA, NaOH solution, 6% (w/v) SDS and 1.875 M Tris pH 8.8 solution) and 1% (v/v) Dithiothreitol, all from Sigma-Aldrich, Portugal). Samples were macerated and denatured for 1 h at 65 °C. For SDS-PAGE, 30 μL equivalents of each sample were loaded in a 4–8% and 4–14% SDS polyacrylamide gel (Sigma-Aldrich, Portugal) and subsequently transferred onto a nitrocellulose membrane (GE Healthcare, UK). For the identification of the proteins of interest, unspecific staining was blocked with 4% (w/v) BSA solution in tris-buffered saline with 0.1% (v/v) Tween 20 (Sigma-Aldrich, Portugal) for 90 min. The blot was incubated overnight at 4 °C with different antibodies (Supplementary Table 4) and with a rabbit polyclonal GAPDH antibody (1:10,000, loading control, Abcam, UK). The bound antibodies were detected with an anti-rabbit/mouse IR680/800Cw secondary antibody (1:15,000, Sigma-Aldrich, Portugal) after 1 h incubation. Proteins were visualized in the 700 or 800 channel of an Odyssey Fc Imaging System (LI-COR, US). Bands were imaged and quantified using the Image Studio Software (LI-COR, US). All blots and gels derived from the same experiment and were processed in parallel.

### Enzyme-linked immunosorbent assay (ELISA)

The levels of VEGF and FGF-2 secreted by the SVF cells in the spongy-like hydrogels were quantified by ELISA assays (R&D Systems, UK) after 7 days of culture. Assays were carried out according to the manufacturer’s instructions and the absorbance of each sample was read at 450 nm using a Synergy HT plate reader (BioTek, USA). The quantity of VEGF and FGF-2 protein was determined against a standard curve.

### Immunocytochemistry

Cell-laden spongy-like hydrogels were fixed with 10% (v/v) Formalin for 24 h at RT and then incubated with 0.2 % (v/v) Triton X-100 (Sigma-Aldrich, Portugal) for 30 min at RT for cell permeabilization. Afterwards, samples were blocked with 3% (w/v) BSA for 1 h and then incubated with anti-human primary antibodies (Supplementary Table 5) diluted in 1% BSA solution in PBS overnight at 4 °C. After washing with PBS, samples were incubated for 1 h at RT with the secondary antibody Alexa Fluor 488 donkey anti-mouse (Life Technologies, CA, USA) at a dilution of 1:500 in1 % BSA solution in PBS. Nuclei were counterstained with DAPI. Constructs were observed using a Leica TCS SP8 confocal microscope (Leica, Germany).

### In vivo assay

The implantation procedure was approved by the *Direcção Geral de Alimentação Veterinária*, the Portuguese National Authority for Animal Health, and all the surgical procedures respected the national regulations and the international animal welfare rules, according to the Directive 2010/63/EU. Athymic nude mice NU(NCr)-Foxn1nu (Charles River, France), 6 weeks old, were used. One full-thickness wound with a 5 mm diameter was created in the back of each mice. Animals were randomly assigned to three groups: (A) GG/GGDVS-RGD spongy-like hydrogels – CTRL; (B) GG/GGDVS-RGD spongy-like hydrogels with freshly isolated SVF cells – SVFfr; (C) GG/GGDVS-RGD spongy-like hydrogels cultured for 7 days with SVF cells – SVFpv. A total of 36 animals, six animals per condition and per time point (5 and 28 days) were used. Mice were anaesthetized with an i.p injection of a mixture of ketamine (75 mg kg^−1^, Imalgene, Merial, France) and metedomidine (1 mg kg^−1^, Domitor, Orion Pharma, Finland). The back of the animals was disinfected with betaisodone and 70% ethanol and a full-thickness skin excision at approximately 0.5 cm caudal to the left scapula was performed using a 5 mm biopsy punch. A donut-shaped 5 mm silicone splint (ATOS Medical, Sweden) was glued and sutured around the wound to minimize wound contraction. After transplantation of the constructs, wounds were successively covered with Tegaderm transparent dressing (3 M, USA), Omnifix (Hartmann, USA), and Leukoplast (Essity, Spain) to avoid any dislocation and to protect the whole treatment set. After surgery, atipamezole (1 mg kg^−1^, Antisedan, Pfizer, Finland) was administered to the animals. The animals were kept separately and received daily analgesia with metamizole (200 μg g^−1^ BW, Nolotil, Boehringer Ingelheim, Germany) in the drinking water for the first 72 h. At each time-point the assigned animals were sacrificed by CO_2_ inhalation and the constructs/tissue were explanted for histological analysis.

### Histological analysis

Explanted tissue was fixed in 10% (v/v) formalin, dehydrated, embedded in paraffin (Thermo Scientific, USA), and cut into 4.5 μm sections. Tissue sections were deparaffinized in xylene, re-hydrated, and boiled for 5 min in Tris-EDTA buffer (10 M Tris Base, 1 mM EDTA solution, and 0.05% (v/v) Tween 20, pH 9) for antigen retrieval. Afterwards, sections were permeabilized with 0.2% Triton X-100 and unspecific staining was blocked with 2.5% Horse Serum (Vector Labs). Primary antibodies (Supplementary Table 5) were incubated overnight at 4 °C. For detection, VECTASTAIN Elite ABC Kit (Vector Labs) was used according to the manufacturer’s instructions. Nuclei were stained with Gill’s haematoxylin. All samples were examined under a Leica DM750 microscope, using LEICA Acquire software.

### Image analysis

Images of five different random fields were acquired for each condition and experiment and used for image analyses. ImageJ software (v2.3.0/1.53m) was used to count the number of PECAM1^+^ cells, in relation to the total number of cells (DAPI), as well as to quantify the number of nodes, junctions, meshes, segments, branches, and segment length of the capillary-like structures using the angiogenesis plug-in^55^.

Images of six different random fields of four non-consecutive tissue sections per time-point and per animal were acquired and used to quantify the number of vessels and respective diameters. Vessels in the implantation site were quantified from the PECAM1 immunostained tissue sections. ImageJ software was used to count the vessels and measure all the vessels diameter for each image. Number of vessels is presented as an average of the counted fields and expressed as number of vessels mm^−2^. Vessel diameter is presented as an average of all measured vessels per condition and expressed in μm.

### Statistical analysis

Statistical analysis was performed using PRISM software (v8.2.1, GraphPad Software Inc., San Diego, USA) for Mac OS X. Shapiro–Wilk test was performed to validate normality of data prior to statistical testing. A one-way or two-way analysis of variance (ANOVA) with a Tukey multiple comparison post-test was used to analyze the results with a normal distribution. Otherwise, data were analyzed with the Kruskal–Wallis test with Dunn’s multiple comparison post-test. Significance was set to 0.05 (95% of confidence interval). All quantitative data refer to 3 independent experiments (*n* = 3) with at least three replicates in each condition in each experiment and are presented as mean ± standard deviation.

### Reporting summary

Further information on research design is available in the Nature Research Reporting Summary linked to this article.

## Supplementary information

Supplementary information

Supplementary Video 1

REPORTING SUMMARY

## Data Availability

All data needed to evaluate the conclusions in the paper are present in the paper and/or the Supplementary Materials. Additional data related to this paper may be requested from the corresponding author.
