# Supplementary material for: Integrin-specific hydrogels for growth factor-free vasculogenesis
Source: NPJ Regen Med. 2022 Sep 27;7:57. doi: 10.1038/s41536-022-00253-4 (PMC9515164; doi:10.1038/s41536-022-00253-4)

Integrin-specific hydrogels for growth factor-free vasculogenesis

Helena R. Moreira[1,2], Daniel B. Rodrigues[1,2], Sara Freitas-Ribeiro[1,2], Lucília P. da Silva[1,2], Alain da S. Morais[1,2], Mariana Jarnalo [3,4], Ricardo Horta[3,4], Rui L. Reis[1,2], Rogério P. Pirraco[1,2], Alexandra P. Marques[1,2,*]

[1] 3B's Research Group, I3Bs – Research Institute on Biomaterials, Biodegradables and Biomimetics, University of Minho, Headquarters of the European Institute of Excellence on Tissue Engineering and Regenerative Medicine, Avepark – Zona Industrial da Gandra, Guimarães 4805-017, Portugal

[2] ICVS/3B's – PT Government Associate Laboratory, Braga/Guimarães 4805-017, Portugal

[3] Department of Plastic and Reconstructive Surgery, and Burn Unity, Centro Hospitalar de São João, Porto, Portugal

[4] Faculty of Medicine - University of Porto, Portugal

* Corresponding author: apmarques@i3bs.uminho.pt

SUPPLEMENTARY INFORMATION

a

Peptide	% GGDVS (w/v)	Conjugation efficiency
RGD	0.25%	95.3 ± 0.9
	0.5%	91.5 ± 1.6
T1	0.25%	89.0 ± 2.3
	0.5%	89.7 ± 1.0

b

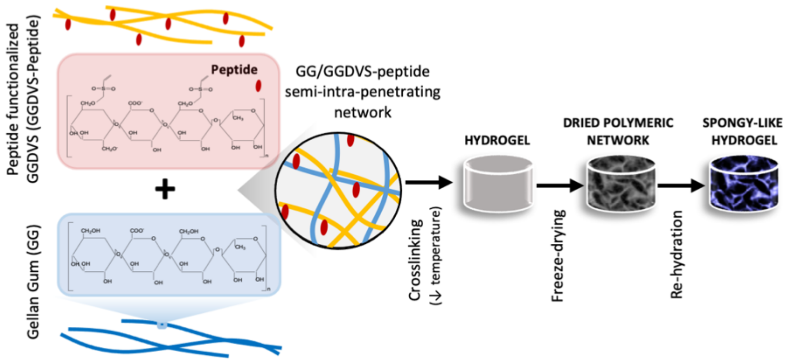

Supplementary Figure 1 - a Peptide conjugation efficiency. **b** Schematic representation of GGDVS functionalization and processing methodology to attain the spongy-like hydrogels. GGDVS is functionalized with the peptide sequences RGD and T1 and combined with unmodified GG at the time of the hydrogel formation obtaining a semi-interpenetrating network. After stabilization of the networks, hydrogels are freeze-dried resulting in dried polymeric structures. Integrin-specific spongy-like hydrogels are obtained after re-hydration of the dried structures.

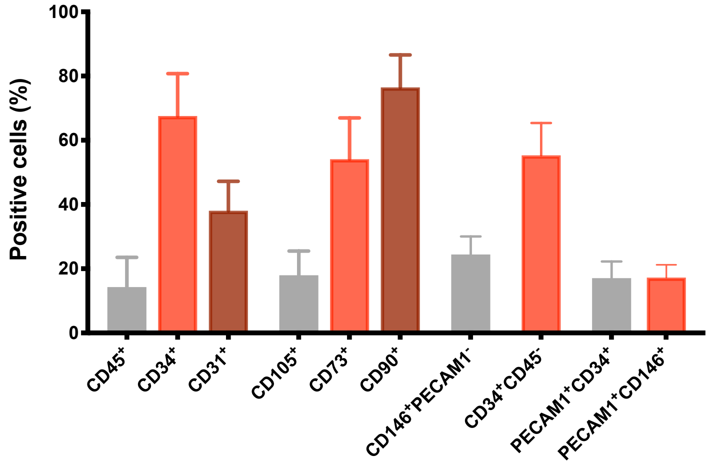

Supplementary Figure 2 - Expression profile of membrane markers of SVF cells after isolation determined by flow cytometry. Markers typically linked to mesenchymal (CD105, CD73 and CD90), pericytic ($CD146^{+}PECAM1^{-}$), endothelial ($PECAM1^{+}CD146^{+}$ and $PECAM1^{+}CD34^{+}$) and endothelial progenitor ($CD34^{+}CD45^{-}$) cells [1] were analysed.

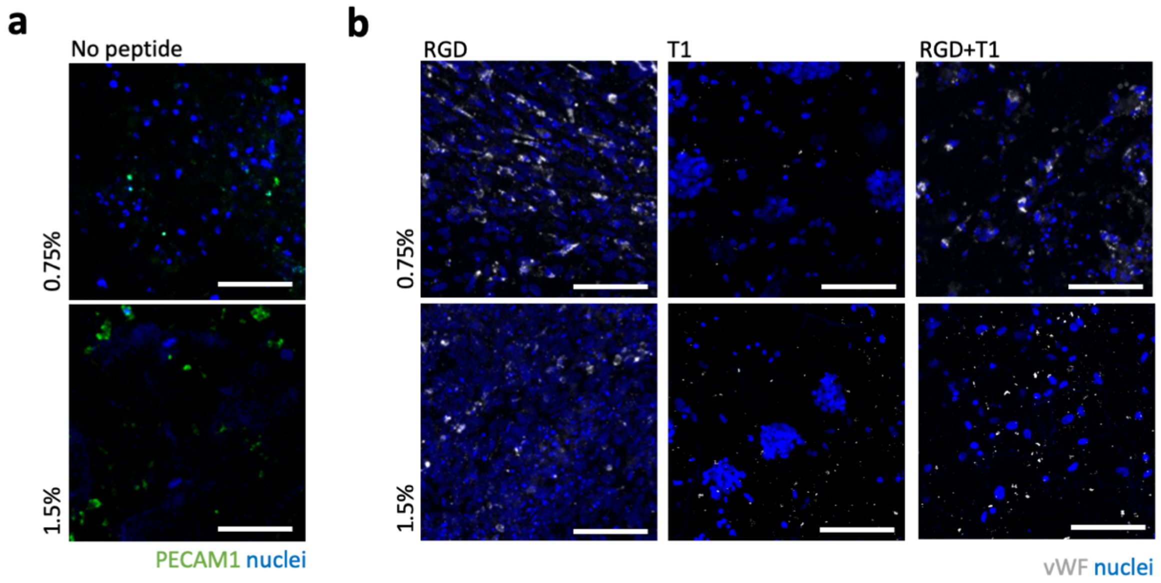

Supplementary Figure 3 - a Representative immunocytochemistry images of SVF cells expressing PECAM1 in the non-functionalized materials after 7 days of culture. Nuclei were counterstained with DAPI. Scale bar = 100 µm. **b** Representative images of the expression of vWF by SVF cells in the functionalized materials after 7 days. Nuclei were counterstained with DAPI. Scale bar = 100 µm.

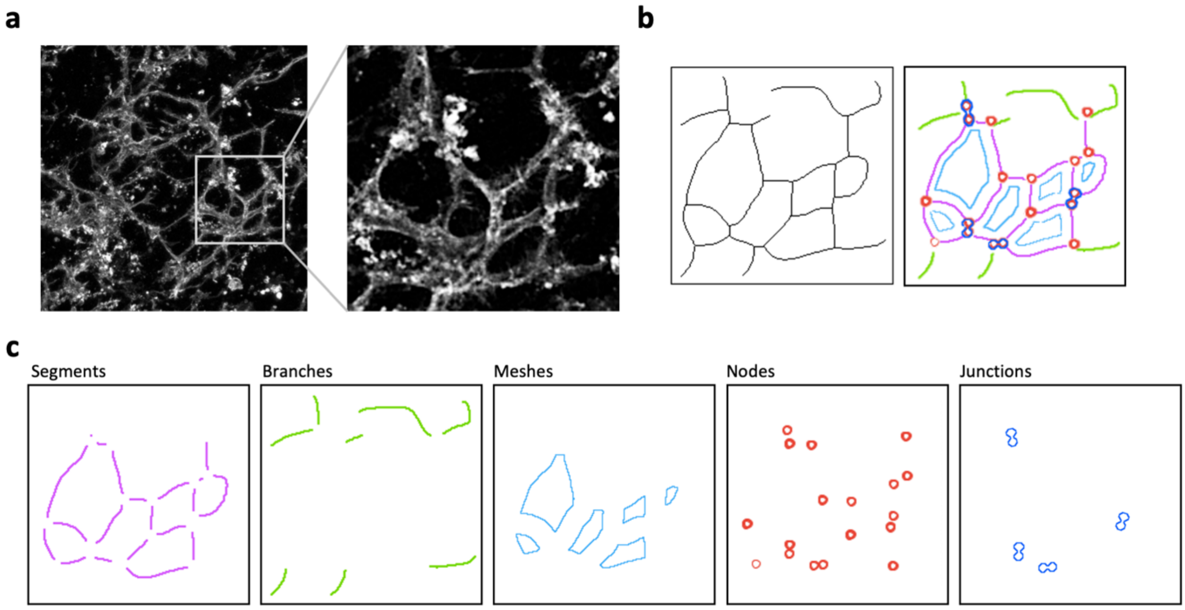

Supplementary Figure 4 – Schematic representation of the measurements obtained from the Angiogenesis Analyzer in ImageJ. a Initial image used for the measurements. **b** Skeleton of the image. **c** Detection of segments (purple) – lines connected by two nodes, branches (green) – lines connected by a node, meshes (ligh blue) – closed areas surrounded by segments, nodes (red) – minimum structure allowing a bifurcation, and junctions (dark blue) – four of more connected segments.

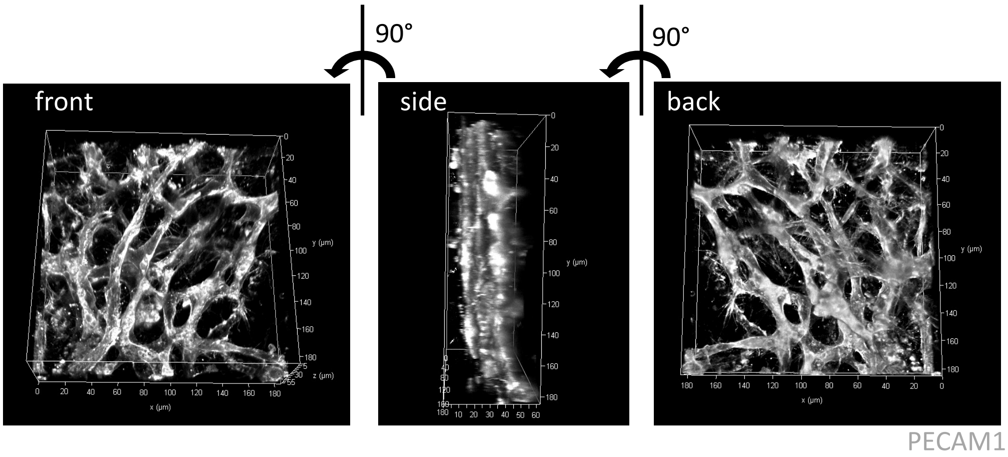

Supplementary Figure 5 - 3D view of a representative PECAM1⁺ vascular-like structure formed in the 0.75 % GG/GGDVS-RGD spongy-like hydrogel after 14 days in culture.

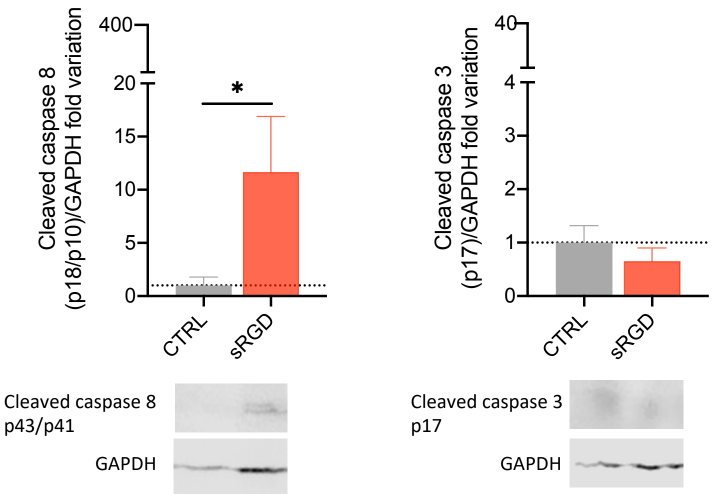

Supplementary Figure 6 - Expression of Caspase 8 and Caspase 3 right after peptide exposure to SVF cells determined by western blot. Plotted data was determined in relation to GAPDH expression. Quantitative results are expressed as the mean ± standard deviation where n = 3, * $p < 0.05$, ** $p < 0.01$, *** $p < 0.001$, **** $p < 0.0001$, two-tailed unpaired t test.

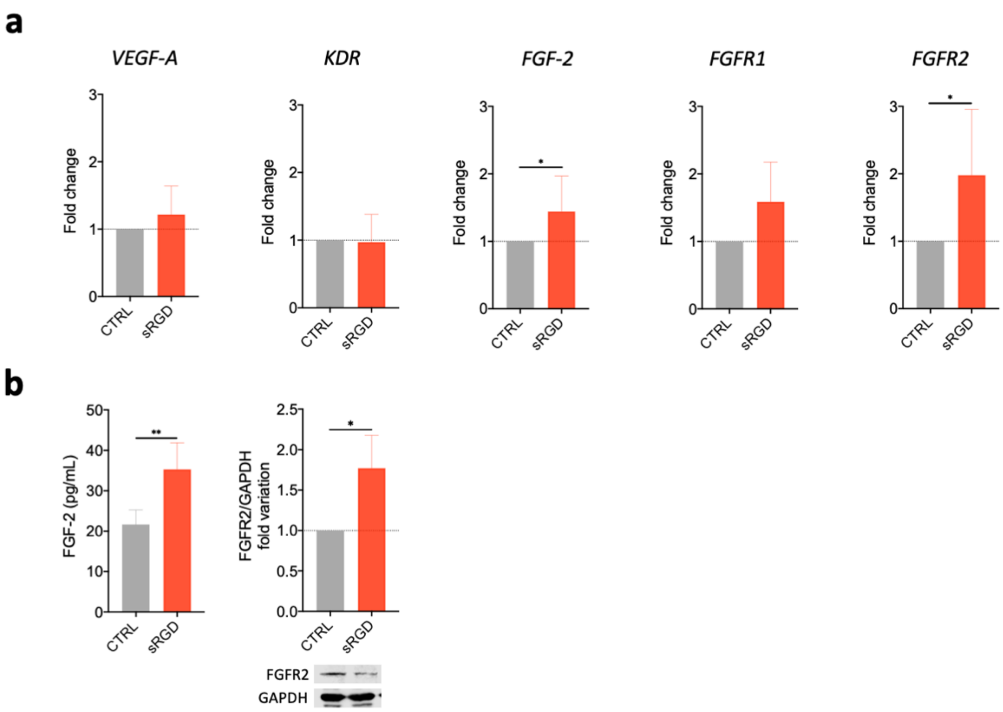

Supplementary Figure 7 - a VEGF, KDR, FGF2, FGFR2 and FGFR1 mRNA expression in SVF cells pre-incubated with RGD peptide and cultured for 7 days in the 0.75 % GG/GGDVS-RGD spongy-like hydrogels. mRNA expression was determined by qPCR using β2M as reference gene and compared to mRNA expression of the Control (0.75 % GG/GGDVS-RGD spongy-like hydrogels with SVF cells after 7 days of culture). **b** FGF-2 secretion and FGFR2 and ERK1/2 protein expression, determined by western blot, in SVF cells pre-incubated with RGD peptide and cultured for 7 days in the 0.75 % GG/GGDVS-RGD spongy-like hydrogels. Plotted western blot data was determined in relation to GAPDH expression. Results are expressed as the mean ± standard deviation where n = 3, * $p < 0.05$, ** $p < 0.01$, *** $p < 0.001$, **** $p < 0.0001$, two-tailed unpaired t test.

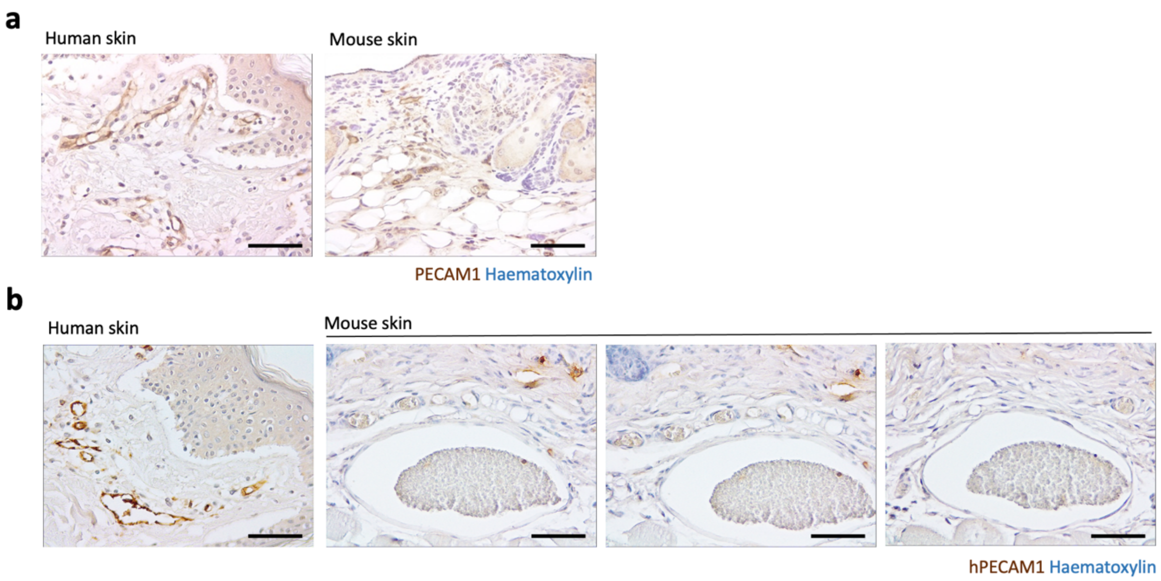

Supplementary Figure 8 - Specificity of anti-PECAM1 antibodies. **a** Representative immunocytochemistry images for the antibody with cross reactivity to human and mouse used to quantify the total number of vessels and determine their diameter. **b** Representative immunocytochemistry images for the anti-human PECAM1 antibody with reactivity to human skin but not to mouse skin (3 consecutive sections) used to determine the number of vessels incorporating human cells. Scale bar = 50 µm.

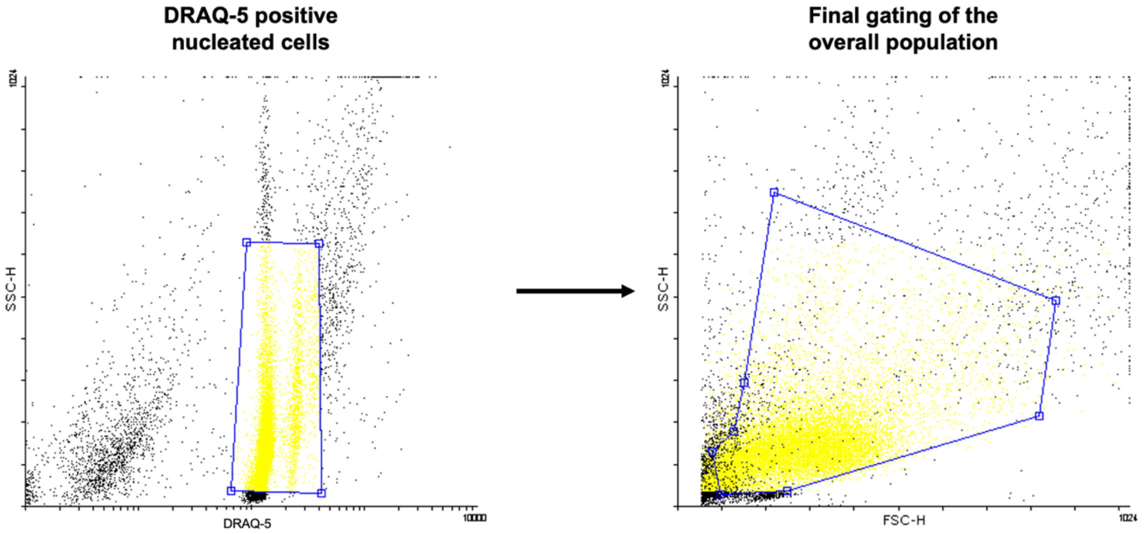

Supplementary Figure 9 – Flow cytometry sequential gating strategy for SVF cells. Debris and eunucleated cells are eliminated by selecting for low SSC, DRAQ-5 positive cells. Final gating of the overall population is then made on the FSC vs SSC plot.

Supplementary Table 1 - GG/GGDVS-peptide(s) formulations

% GG (w/v)	% GGDVS (w/v)	Peptide(s)	Name
0.5	0.25	RGD	0.75%RGD
		T1	0.75%T1
		RGD+T1	0.75%RGD+T1
1	0.5	RGD	1.5%RGD
		T1	1.5%T1
		RGD+T1	1.5%RGD+T1

Supplementary Table 2 - Flow cytometry antibodies

Antibody	Brand	Ref	Dilution	Host
CD105-FITC	Bio-Rad	MCA1557F	1 μl per 10^5 cells	Ms
CD73-PE	BD Biosciences, USA	550257	10 μl per 10^5 cells	Ms
CD90-APC	BD Biosciences, USA	559869	1 μl per 10^5 cells	Ms
CD45-FITC	BD Biosciences, USA	555482	10 μl per 10^5 cells	Ms
CD31/PECAM1-APC	R&D Systems, USA	FAB3567A	1 μl per 10^5 cells	Ms
CD34-PE	BD Biosciences, USA	555822	10 μl per 10^5 cells	Ms
CD146-PE	BD Biosciences, USA	561013	10 μl per 10^5 cells	MS

Legend: Ms - mouse

Supplementary Table 3 - Primers sequences

Gene and accession number	Sequence (5'-3')	Annealing temperature (ºC)
Human β2M NM_004048.4	TGGAGGCTATCCAGCGTACT	59.5
	CGGATGGATGAAACCCAGACA	
Human VEGFA NM_ 003376.6	GATCCGCAGACGTGTAAATG	59
	CCCTCCCAACTCAAGTCCAC	
Human KDR NM_002253.4	CTAGGTGCCTGTACCAAGCC	59
	GCCCCTTTGGTCTTGTAGGG	
Human FLT-1 NM_002019.4	CGGACTGTGGCTGTGAAAATG	59.8
	TGGCCAATGTGGGTCAAGAT	
Human FGF2 NM_001361665.2	CACCTATAATTGGTCAAAGTGG	59
	CAGAAATTCAGTAGATGTTTCCC	
Human FGFR1 NM_023110.3	ACCAAACCGTATGCCCGTAG	57.5
	CCCACTGGAAGGGCATTTGA	
Human FGFR2 NM_000141.5	CCTCTCGTTCCCCAAATC	59
	GAGTGGTCCTTGGGTCTT	
Human ANGPT1 NM_001146.5	TGCAGAGAGATGCTCCACAC	56.6
	TCTCAAGTTTTTGCAGCCACT	
Human TEK NM_000459.5	TGCGAGATGGATAGGGCTTG	59.2
	AGGATGGGAAAGGCTGTATCTT	
Human PECAM1 NM_000442.5	GAGGGGCCACATGCATCTAT	59.7
	AGACCTGCTCGGTTCTCTCT	
Human VWF NM_000552.5	CCCTGGGTTACAAGGAAGAAAAT	59
	AGTGTCATGATCTGGCCTCCTCTTAG	

Supplementary Table 4 - Western blot antibodies

Antibody	Brand	Ref	Dilution	Host
FAK	Abcam, UK	ab40794	1:500	Rb
Phospho-FAK	Cell Signaling, USA	1673283S	1:1000	Rb
AKT1	Cell Signaling, USA	9272S	1:1000	Rb
Phospho-AKT	Abcam, UK	ab81283	1:1000	Rb
ERK 1+2	Abcam, UK	ab17942	1:1000	Rb
Phospho-ERK 1+2	Abcam, UK	ab50011	1:1000	Ms
Paxillin	Abcam, UK	ab32084	1:5000	Rb
Talin 1+2	Abcam, UK	ab11188	1:1000	Ms
Vinculin	Sigma, Portugal	V9131	1:200	Ms
KDR	Abcam, UK	ab39256	1:500	Rb
FGFR2	Abcam, UK	ab10648	1:1000	Rb
Caspase 8	Santa Cruz Biotechnology, USA	sc-81656	1:200	Ms
Caspase 3	Abcam, UK	ab32351	1:1000	Rb
GAPDH	Abcam, UK	ab181602	1:10000	Rb

Legend: Rb – rabbit, Ms - mouse

Supplementary Table 5 - Immunolabelling antibodies

	Antibody	Brand	Ref	Dilution	Host
**ICC**	PECAM1 (CD31)	Dako, Denmark	M0823	1:30	Ms
	von Willbrand factor (vWF)	Abcam, USA	ab201336	1 μg mL^{-1}	Ms
	Fibronectin	Abcam, UK	ab2413	1:100	Rb
	Laminin	Abcam, USA	ab11575	1:30	Rb
	Collagen type IV	Abcam, USA	ab6586	1:100	Rb
	VE-cadherin	Abcam, USA	ab33168	1 μg $mL\text{-}^{1}$	Rb
**IHC**	human PECAM1	Dako, Denmark	M0823	1:30	Ms
	total PECAM1	Abcam	ab28364	1:25	Rb

Legend: ICC – immunocytochemistry, IHC – immunohistochemistry, Rb – rabbit, Ms - mouse

SUPPLEMENTARY REFERENCES

[1] K. Yoshimura, T. Shigeura, D. Matsumoto, T. Sato, Y. Takaki, E. Aiba-Kojima, K. Sato, K. Inoue, T. Nagase, I. Koshima, K. Gonda, Characterization of freshly isolated and cultured cells derived from the fatty and fluid portions of liposuction aspirates, J. Cell. Physiol. 208 (2006) 64–76. https://doi.org/10.1002/jcp.20636.

Uncropped original scans

Fig. 3c, Supplementary Fig. 6

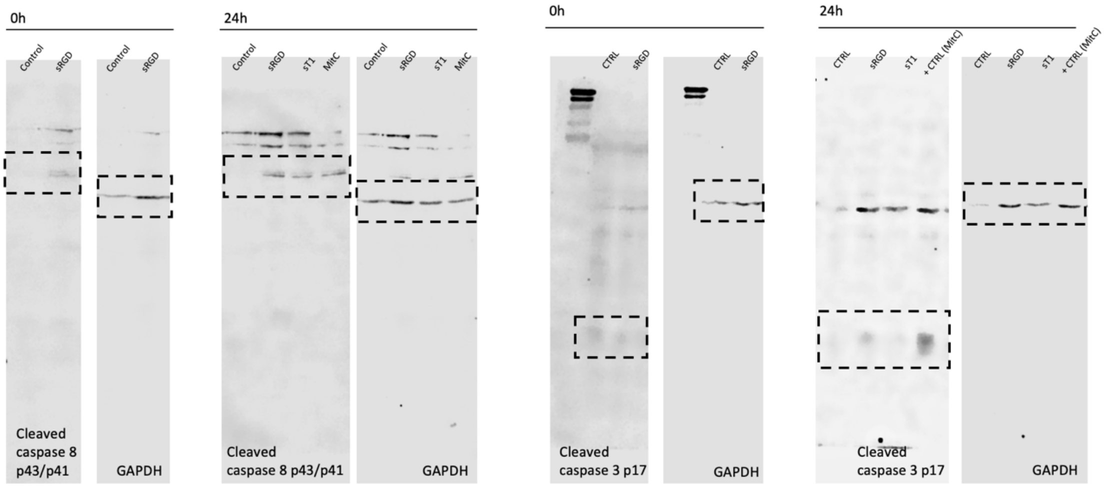

Fig. 4b,c,d, Supplementary Fig. 7b

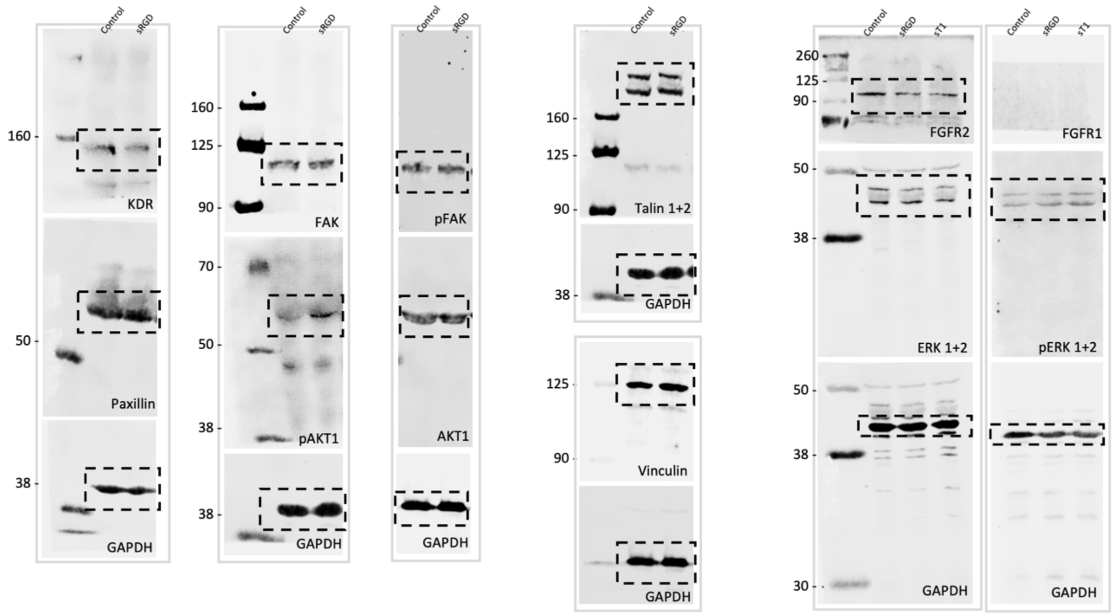

Supplement: Supplementary file 1 — Supplementary information [file 41536_2022_253_MOESM1_ESM.pdf]
