# Supplementary material for: Integrin-specific hydrogels for growth factor-free vasculogenesis
Source: NPJ Regen Med. 2022 Sep 27;7:57. doi: 10.1038/s41536-022-00253-4 (PMC9515164; doi:10.1038/s41536-022-00253-4)
Supplement: Supplementary file 3 — REPORTING SUMMARY [file 41536_2022_253_MOESM3_ESM.pdf]

nature portfolio

Corresponding author(s): Alexandra P. Marques

Last updated by author(s): Apr 13, 2022

Reporting Summary

Nature Portfolio wishes to improve the reproducibility of the work that we publish. This form provides structure for consistency and transparency in reporting. For further information on Nature Portfolio policies, see our Editorial Policies and the Editorial Policy Checklist.

Statistics

For all statistical analyses, confirm that the following items are present in the figure legend, table legend, main text, or Methods section.

n/a	Confirmed	
☐	☒	The exact sample size (*n*) for each experimental group/condition, given as a discrete number and unit of measurement
☐	☒	A statement on whether measurements were taken from distinct samples or whether the same sample was measured repeatedly
☐	☒	The statistical test(s) used AND whether they are one- or two-sided<br>*Only common tests should be described solely by name; describe more complex techniques in the Methods section.*
☒	☐	A description of all covariates tested
☐	☒	A description of any assumptions or corrections, such as tests of normality and adjustment for multiple comparisons
☐	☒	A full description of the statistical parameters including central tendency (e.g. means) or other basic estimates (e.g. regression coefficient) AND variation (e.g. standard deviation) or associated estimates of uncertainty (e.g. confidence intervals)
☐	☒	For null hypothesis testing, the test statistic (e.g. *F*, *t*, *r*) with confidence intervals, effect sizes, degrees of freedom and *P* value noted<br>*Give P values as exact values whenever suitable.*
☒	☐	For Bayesian analysis, information on the choice of priors and Markov chain Monte Carlo settings
☒	☐	For hierarchical and complex designs, identification of the appropriate level for tests and full reporting of outcomes
☒	☐	Estimates of effect sizes (e.g. Cohen's *d*, Pearson's *r*), indicating how they were calculated

Our web collection on statistics for biologists contains articles on many of the points above.

Software and code

Policy information about availability of computer code

Data collection	Micro-computed tomography: SkyScan 1072 scanner (SkyScan, Kontich, Belgium)<br>Flow cytometry: BD FACSCalibur (BD)<br>2D Brightfield/Fluoresence microscopy: Axio Observer inverted Microscope (Zeiss, Germany), Leica DM750 microscope (Leica, Germany)<br>3D Fluoresence microscopy: Leica TCS SP8 confocal microscope (Leica, Germany)<br>qPCR: NanoDrop N-1000 Spectrophotometer (Thermo Fischer Scientific, USA), MasterCycler Realplex4 (Eppendorf, Germany)<br>Western blot: Odyssey Fc Imaging System (LI-COR, US)<br>ELISA: Synergy HT plate reader (BioTek, USA)
Data analysis	Micro-computed tomography: CT Analyzer v1.5.1.5 (SkyScan).<br>Flow cytometry: Cyflogic v1.2.1<br>Image analysis: ZEN Blue 2012 software (Zeiss, Germany), LEICA Acquire software (Leica, Germany), FIJI for ImageJ v2.3.0<br>Statistical analysis: GraphPad PRISM v8.2.1 for Mac (GraphPad Software Inc., CA, USA)

For manuscripts utilizing custom algorithms or software that are central to the research but not yet described in published literature, software must be made available to editors and reviewers. We strongly encourage code deposition in a community repository (e.g. GitHub). See the Nature Portfolio guidelines for submitting code & software for further information.

Data

Policy information about availability of data

All manuscripts must include a data availability statement. This statement should provide the following information, where applicable:

- Accession codes, unique identifiers, or web links for publicly available datasets
- A description of any restrictions on data availability
- For clinical datasets or third party data, please ensure that the statement adheres to our policy

All data needed to evaluate the conclusions in the paper are present in the paper and/or the Supplementary Materials. Additional data related to this paper may be requested from the authors.

Field-specific reporting

Please select the one below that is the best fit for your research. If you are not sure, read the appropriate sections before making your selection.

☒ Life sciences ☐ Behavioural & social sciences ☐ Ecological, evolutionary & environmental sciences

For a reference copy of the document with all sections, see nature.com/documents/nr-reporting-summary-flat.pdf

Life sciences study design

All studies must disclose on these points even when the disclosure is negative.

Sample size	Required experimental sample sizes were estimates based on previos established protocols in the field of tissue engineering. The sample sizes were adquate as the differences between experimental groups were reproducible. All n values are clearly indicated within the figure legends.
Data exclusions	No data was excluded from the analysis.
Replication	Experiments included multiple independent biological donors and key experiments were repeated to ensure replicability. All attempts at replication were successful and all data is presented in the manuscript.
Randomization	Animals were randomized to treatment groups.
Blinding	Blinding to group allocation was not possible in animal studies. However, automated analyses were used to minimize the effects of investigator bias.

Reporting for specific materials, systems and methods

We require information from authors about some types of materials, experimental systems and methods used in many studies. Here, indicate whether each material, system or method listed is relevant to your study. If you are not sure if a list item applies to your research, read the appropriate section before selecting a response.

Materials & experimental systems

n/a	Involved in the study	
☐	☒	Antibodies
☐	☒	Eukaryotic cell lines
☒	☐	Palaeontology and archaeology
☐	☒	Animals and other organisms
☒	☐	Human research participants
☒	☐	Clinical data
☒	☐	Dual use research of concern

Methods

n/a	Involved in the study	
☒	☐	ChIP-seq
☐	☒	Flow cytometry
☒	☐	MRI-based neuroimaging

Antibodies

Antibodies used	For flow cytometry:<br>CD105-FITC, Bio-Rad, Ref #MCA1557F<br>CD73-PE, BD Biosciences, USA, Ref #550257<br>CD90-APC, BD Biosciences, USA, Ref #559869<br>CD45-FITC, BD Biosciences, USA, Ref #555482<br>CD31/PECAM1-APC, R&D Systems, USA, Ref #FAB3567A<br>CD34-PE, BD Biosciences, USA, Ref #555822<br>CD146-PE, BD Biosciences, USA, Ref #561013

For western blot:
FAK, Abcam, UK, Ref #ab40794 (1:500)
Phospho-FAK, Cell Signaling, USA, Ref #1673283S (1:1000)
AKT1, Cell Signaling, USA, Ref #9272S (1:1000)
Phospho-AKT, Abcam, UK, Ref #ab81283 (1:1000)
ERK 1+2, Abcam, UK, Ref #ab17942 (1:1000)
Phospho-ERK 1+2, Abcam, UK, Ref #ab50011 (1:1000)
Paxillin, Abcam, UK, Ref #ab32084 (1:5000)
Talin 1+2, Abcam, UK, Ref #ab11188 (1:1000)
Vinculin, Sigma, Portugal, Ref #V9131 (1:200)
KDR, Abcam, UK, Ref #ab39256 (1:500)
FGFR2, Abcam, UK, Ref #ab10648 (1:1000)
Caspase 8, Santa Cruz Biotechnology, USA, Ref #sc-81656 (1:200)
Caspase 3, Abcam, UK, Ref #ab32351 (1:1000)
GAPDH, Abcam, UK, Ref #ab181602 (1:10000)

For immunocytochemistry/immunohistochemistry:
PECAM1 (CD31), Dako, Denmark, Ref #M0823 (1:30)
von Willbrand factor (vWF), Abcam, USA, Ref #ab201336 (1 ug/mL)
Fibronectin, Abcam, UK, Ref #ab2413 (1:100)
Laminin, Abcam, USA, Ref #ab11575 (1:30)
Collagen type IV, Abcam, USA, Ref #ab6586 (1:100)
VE-cadherin, Abcam, USA, Ref #ab33168 (1 ug/mL)
human PECAM1, Dako, Denmark, Ref #M0823 (1:30)
total PECAM1, Abcam ab28364 (1:25)

Validation

All antibodies used in this manuscript have been previously validated in the literature and used as manufacturers' provided data sheets. Human PECAM1 (M0823, Dako) was validated in this manuscript has shown in supplementary figure 8.

Eukaryotic cell lines

Policy information about cell lines

Cell line source(s)

SVF cells were harvested form adipose tissue from skin specimens of healthy donors undergoing abdominoplasties after written informed consent and under the protocol established and approved between the Ethical Committees of Hospital S. João (Porto, Portugal) (Nr 477/2020) and University of Minho (CEICVS Nr 135/2020).

Authentication

N/A

Mycoplasma contamination

All cells were regularly tested and were always negative for mycoplasma.

Commonly misidentified lines
(See ICLAC register)

N/A

Animals and other organisms

Policy information about studies involving animals; ARRIVE guidelines recommended for reporting animal research

Laboratory animals

Athymic nude mice NU(NCr)-Foxn1nu (Charles River, France), 6 weeks old, were used.

Wild animals

The study did not involve wild animals.

Field-collected samples

The study did not involve samples collectes from the field.

Ethics oversight

The animal study was approved by the Direcção Geral de Alimentação Veterinária (DGAV), the Portuguese National Authority for Animal Health, and all the surgical procedures respected the national regulations and the international animal welfare rules, according to the Directive 2010/63/EU.

Note that full information on the approval of the study protocol must also be provided in the manuscript.

Flow Cytometry

Plots

Confirm that:

☒ The axis labels state the marker and fluorochrome used (e.g. CD4-FITC).

☐ The axis scales are clearly visible. Include numbers along axes only for bottom left plot of group (a 'group' is an analysis of identical markers).

☐ All plots are contour plots with outliers or pseudocolor plots.

☒ A numerical value for number of cells or percentage (with statistics) is provided.

Methodology

Sample preparation	Sample preparation was carried out as detailed in the Methods section.
Instrument	BD FACSCalibur
Software	Cyflogic v1.2.1
Cell population abundance	N/A
Gating strategy	Cells were gated on FSC/SSC first. Afterwards, cells were gated on DRAQ5 (eBioscoence, USA) for positive nuclei, to discern the cells of interest from the any remaining erythrocytes and tissue debris.

☐ Tick this box to confirm that a figure exemplifying the gating strategy is provided in the Supplementary Information.
